# A Bifunctional Iron‐Nickel Oxygen Reduction/Oxygen Evolution Catalyst for High‐Performance Rechargeable Zinc–Air Batteries

**DOI:** 10.1002/smll.202409161

**Published:** 2024-11-27

**Authors:** Zhengfan Chen, Weiyi Cheng, Kecheng Cao, Meng Jin, Sarra Rahali, Soressa Abera Chala, Elnaz Ebrahimi, Nana Ma, Rongji Liu, Keseven Lakshmanan, Chia‐Yu Chang, Chun‐Chi Cheung, Haojian Luo, Yongkang Wang, Bing Joe Hwang, Carsten Streb

**Affiliations:** ^1^ Department of Chemistry Johannes Gutenberg University Mainz Duesbergweg 10–14 55128 Mainz Germany; ^2^ School of Chemistry and Chemical Engineering Henan Normal University Xinxiang 453007 China; ^3^ School of Physical Science and Technology Shanghai Tech University Shanghai 201210 China; ^4^ Sustainable Electrochemical Energy Development (SEED) Center National Taiwan University of Science and Technology Taipei 106335 Taiwan; ^5^ Department of Molecular Spectroscopy Max Planck Institute for Polymer Research 55128 Mainz Germany

**Keywords:** density functional theory, electrocatalysis, oxygen evolution reaction, oxygen reduction reaction, Zinc–Air–Battery

## Abstract

Efficient and robust electrocatalysts for the oxygen reduction reaction (ORR) and oxygen evolution reaction (OER) are crucial for fuel cells, metal‐air batteries, and other energy technologies. Here, a highly stable, efficient bifunctional OER/ORR electrocatalyst (FeNi‐NC@MWCNTs) is reported and demonstrated its integration and robust performance in an aqueous Zinc–air battery (ZAB). The catalyst is based on neighboring iron/nickel sites (FeNiN_6_) which are atomically dispersed on porous nitrogen‐doped carbon particles. The particles are wrapped in electrically conductive multi‐walled carbon nanotubes for enhanced electrical conductivity. Electrocatalytic analyses show high OER and ORR performance (OER/ORR voltage difference = 0.69 V). Catalyst integration in a ZAB results in excellent performance metrics, including an open circuit voltage of 1.44 V, a specific capacity of 782 mAh g^−1^ (at *j* = 15 mA cm^−2^), a peak power density of 218 mW cm^−2^ (at *j* = 260 mA cm^−2^) and long‐term durability over 600 charge/discharge cycles. Combined experimental and theoretical (density functional theory) analyses provide an in‐depth understanding of the physical and electronic structure of the catalyst and the role of the FeNi dual atom reaction site. The study therefore provides critical insights into the structure and reactivity of high‐performance bifunctional OER/ORR catalysts based on atomically dispersed non‐critical metals.

## Graphical abstract

A bifunctional Ni‐Fe dual‐site composite electrocatalyst for the oxygen reduction reaction and oxygen evolution reaction is reported together with the catalyst deployment in a high‐performance rechargeable zinc–air battery.

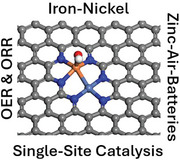

## 1 Introduction

The development of cost‐effective, sustainable, and environmental‐friendly energy conversion and storage devices is a key path toward carbon neutrality.^[^ ^1^, ^2^, ^3^ ^]^ Rechargeable zinc–air batteries (ZABs) are amongst the most promising battery technologies as they combine high energy density (theoretical limit: 1086 Wh kg^−1^), economic viability, and feasible scale‐up.^[^ ^4^, ^5^, ^6^ ^]^ However, the sluggish kinetics of the oxygen reduction reaction (ORR) and the oxygen evolution reaction (OER) result in high overpotentials and unsatisfactory efficiency, thereby causing major roadblocks in the development of ZABs.^[^ ^7^, ^8^ ^]^ While noble metal‐based catalysts might address some of these issues, their scarcity and high cost prohibit large‐scale commercialization.^[^ ^9^, ^10^ ^]^ Thus, the development of highly efficient and robust bifunctional ORR/OER electrocatalysts based on earth‐abundant elements is a grand challenge in sustainable energy research.^[^ ^11^ ^]^

Recently, single‐atom catalysts (SACs) have emerged as promising alternatives to noble metal catalysts.^[^ ^12^, ^13^ ^]^ In SACs, transition metal atoms (especially Fe, Co, Ni, and Mn) are anchored on electroactive matrices, e.g. carbon, often by coordination with dopant elements such as nitrogen, resulting in various M‐N‐C species.^[^ ^14^, ^15^ ^]^ These materials offer unique advantages including strong metal‐substrate interactions,^[^ ^16^ ^]^ tunable electronic structure of the active metal site as well as high electric conductivity, often resulting in excellent redox‐catalytic performance.^[^ ^17^, ^18^, ^19^ ^]^ Also, the nearly molecular design of these materials enables optimum metal‐atom utilization and ‐rational active site design at the atomic level.^[^ ^19^, ^20^ ^]^

However, particularly for ORR, monometallic SACs (often based on Fe, Co) are limited by unfavorable energetics for the binding of key oxygen intermediates (*O_2_ and *OOH), resulting in high O_2_ activation barriers.^[^ ^21^ ^]^ In addition, monometallic SACs often show high overpotentials for the OER reaction, as water oxidation is favored at polynuclear species.^[^ ^22^ ^]^

One promising strategy to overcome these challenges is the introduction of a second metal center, resulting in dual atom catalysts (DACs).^[^ ^23^ ^]^ Compared with SACs, the DACs comprise adjacent hetero‐metal pairs (*e.g*., Fe‐Co, Co‐Mn, Co‐Ni),^[^ ^24^, ^25^, ^26^ ^]^ which exhibit asymmetric charge distribution at the active sites. This feature offers a promising approach to enhance the adsorption and desorption of oxygen intermediates, thereby lowering the energy barriers for O_2_ activation. Moreover, synergistic effects at the dual‐metal site can enhance overall catalytic performance for both ORR and OER.^[^ ^23^, ^27^ ^]^ Despite major efforts in the field, the development of bifunctional DACs for use in ZABs is still in its infancy, and current challenges include control over stability and tuning of the precise coordination environment.^[^ ^28^ ^]^

To date, 2D‐ and 3D‐structured nitrogen‐doped carbon matrices derived from metal‐organic frameworks (MOFs) are often used for anchoring SACs. While these materials offer facile synthetic access, they often result in active sites hidden within the composite, while metal aggregation and particle migration during operation negatively affect mass transfer during operation.^[^ ^29^, ^30^ ^]^ Thus, structural and morphological engineering of nitrogen‐doped carbon substrates is of great significance to fully expose active sites and ensure efficient electron and mass transfer.

To overcome these challenges, SAC composites have been combined with multi‐walled carbon nanotubes (MWCNTs). MWCNTs feature 1D structures with outstanding chemical and thermal stability, high specific surface area, and excellent electrical conductivity, making them ideal substrates for bifunctional ORR/OER electrocatalysts.^[^ ^31^, ^32^ ^]^ In one outstanding example, Masa and co‐workers reported oxygen‐functionalized MWCNTs as substrate for trimetallic (Mn‐Fe‐Ni) oxide nanoparticles, exhibiting remarkable ORR and OER performances.^[^ ^33^ ^]^ Also, Streb, Liu, and coworkers used a facile one‐pot assembly synthetic route to deposit nanostructured manganese vanadium oxides on nitrogen‐sulfur functionalized MWCNTs, which showed excellent ORR and OER performance.^[^ ^34^, ^35^ ^]^ However, establishing a chemical and electronic linkage between the reactive sites and charge transfer materials (MWCNTs) remains challenging, and often results in poor interfacial conductivity and poor long‐term stability. Recently, Sun and colleagues reported an atomically dispersed Fe‐N‐C catalyst fabricated from a Fe modified zeolitic imidazolate framework (ZIF‐8), which demonstrated impressive performance for both ORR and ZABs.^[^ ^36^ ^]^ Liu and collaborators also employed ZIF‐8 as a template and successfully prepared a bifunctional FeCo‐NC catalyst for ORR and OER, enabling stable long‐term ZAB operation.^[^ ^21^ ^]^

Here, we build on these pioneering studies and aim at designing bifunctional ORR/OER composites where ZIF‐derived DACs on conductive N‐doped carbon are hierarchically integrated with MWCNTs to harness reactivity and stability synergisms. We proposed a strategy for anchoring FeNi dual‐metal sites on nitrogen‐doped carbon enwrapped by conductive MWCNTs via a simple two‐step process (denoted as FeNi‐NC@MWCNT). The resulting catalyst demonstrated excellent bifunctional OER and ORR reactivity, outperforming the benchmark Pt/C + IrO_2_ and various related reference catalysts. Complementary experimental and theoretical analyses provided unique information on the reaction mode and synergism of these dual sites, offering distinct reaction pathways that are energetically not accessible by single metal site reference catalysts. The practical applicability and stability of the catalyst were demonstrated by integration in an aqueous rechargeable ZAB which showed excellent performance in long‐term cycling tests.

## 2 Results and Discussion

### 2.1 Catalysts Synthesis and Structural Characterization

**Figure 1:**
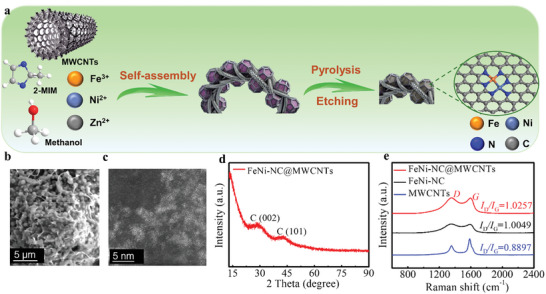
Synthesis, structure, and morphology of the reported catalysts. a) Illustration of the synthesis process of FeNi‐NC@MWCNT. b) SEM image, c) HAADF‐STEM image of FeNi‐NC@MWCNT. d) Powder XRD pattern of FeNi‐NC@MWCNT, and e) Raman spectra of FeNi‐NC@MWCNT, FeNi‐NC, and MWCNTs.

**Figure** **1**a illustrates the two‐step synthetic strategy for the dual‐metal catalyst. First, the precursor FeNi/ZIF‐8@MWCNTs was fabricated by in situ functionalization of ZIF‐8 with Fe^3+^ and Ni^2+^.^[^ ^37^ ^]^ The resulting dodecahedral ZIF‐8 particles were anchored to the surface of MWCNTs, resulting in a self‐assembled macroporous 3D structure. Calcination of this precursor at 950 °C for 3 h under Argon flow gave the target composite, hereafter referred to as FeNi‐NC@MWCNT. Note that during calcination, the precursor particles were converted to 2D graphene‐like nanosheets, which were mechanically stabilized by the interlinking MWCNTs. Also note that the Zn^2+^ metal present in the original ZIF‐8 was removed from the sample by evaporation during pyrolysis, contributing to the formation of a highly porous N‐doped carbon matrix.^[^ ^18^ ^]^ After pyrolysis, the composite was washed repeatedly with aqueous HCl (1.5 M) and deionized water to remove any metal salts and metal nanoparticles.^[^ ^38^ ^]^ To understand the impact of the dual Fe/Ni metal sites, we also synthesized the monometallic Fe‐NC@MWCNTs and Ni‐NC@MWCNTs as reference samples. Also, to assess the impact of the MWCNTs, we fabricated FeNi/ZIF‐8 composites in the absence of MWCNTs.

The morphology and structure of the catalysts were investigated by scanning electron microscopy (SEM) and transmission electron microscopy (TEM). Figure 1b shows the SEM image of FeNi‐NC@MWCNT, the structure of ZIF‐8 nanoparticles was well maintained during Fe/Ni functionalization and pyrolysis. The ZIF‐derived nanoparticles are interspersed within the MWCNTs, resulting in a hierarchical porous structure. Note that the MWCNT‐free reference FeNi‐NC shows similar particle features (Figure S1, Supporting Information), indicating the introduction of MWCNTs does not affect catalyst morphology. Also, SEM and TEM analyses do not indicate any metal or metal oxide nanoparticles in both FeNi‐NC@MWCNT and FeNi‐NC (Figures S2, S3, Supporting Information).

To probe the dispersion of the Fe and Ni sites, we used aberration‐corrected high‐angle annular dark‐field scanning TEM (AC‐HAADF‐STEM). As shown in Figure 1c, a large number of uniformly distributed bright spots are clearly identified in FeNi‐NC@MWCNT, which are assigned to isolated single Fe and/or Ni atoms and/or dual single atoms sites (FeNi sites). The corresponding element mapping analysis confirms the uniform dispersion of Fe, Ni, C, and N in the carbon matrix derived from ZIF‐8 (Figure S4, Supporting Information). Also, inductively coupled plasma optical emission spectroscopy (ICP OES) was used to determine the mass percentage of Fe (0.57 wt.%) and Ni (0.62 wt.%) in FeNi‐NC@MWCNT (Table S1, Supporting Information). Figure 1d represents the powder X‐ray diffraction (pXRD) pattern of FeNi‐NC@MWCNT, the profile shows two broad diffraction peaks at ≈30° and 42° attributed to the (002) and (101) lattice planes of graphitic carbon.^[^ ^20^ ^]^ No peaks of crystalline Fe or Ni metal‐based particles were observed in FeNi‐NC@MWCNT, which is in line with the microscopic analyses. The Raman spectrum of FeNi‐NC@MWCNT (Figure 1e) primarily shows two characteristic bands with the D band at ≈1350 cm⁻¹ and the G band at ≈1595 cm⁻¹. The D band represents defects or/ disorders in the carbon lattice, while the G band corresponds to ordered sp^2^‐hybridized graphitic carbon atoms. The ratio between the D band and G band (I_D_/I_G_) for FeNi‐NC@MWCNT is higher than for the reference samples (see Figure S6, Supporting Information), indicating the introduction of more structural defects during synthesis. The specific surface area and pore size distribution of the catalysts were measured by N_2_ sorption (Figure S7, Supporting Information). Analysis of the data showed that the introduction of MWCNTs significantly increased the specific surface area of FeNi‐NC@MWCNT. Also, FeNi‐NC@MWCNT contains more micropores and mesopores than the FeNi‐NC reference, which can alter mass transfer (particularly of gaseous reagents) within the composite.

**Figure 2:**
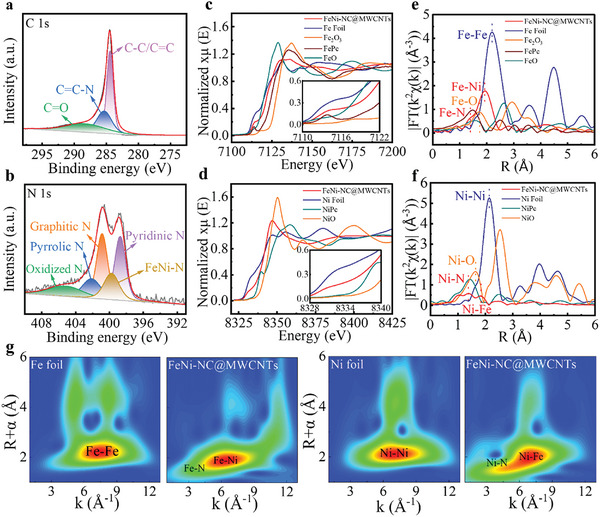
Chemical state and local atomic environment of FeNi‐NC@MWCNT. a) Deconvoluted XPS spectrum for C 1s. b) Deconvoluted XPS spectrum of N 1s. c) Fe K‐edge XANES spectra. d) Ni K‐edge XANES spectra. e) Fourier‐transformed k^2^‐weighted EXAFS spectra of Fe K‐edge. f) Fourier‐transformed k^2^‐weighted EXAFS spectra of Ni K‐edge. g) Wavelet‐transform (WT)‐EXAFS plots for FeNi‐NC@MWCNT, Fe foil, and Ni foil.

X‐ray photoelectron spectroscopy (XPS) measurements were performed to investigate the surface chemical states of the synthesized catalysts. XPS spectra of FeNi‐NC@MWCNT and FeNi‐NC both exhibit distinctive signals of C, N, and O elements, but only very weak Fe and Ni signals due to the low loading. As shown in **Figure** **2**a, the high‐resolution C 1s XPS spectrum of FeNi‐NC@MWCNT is deconvoluted into three peaks at binding energies of 284.5, 285.2, and 288.7 eV, which are assigned to C─C/C═C, C═C─N, and C═O, respectively. The observation of C─N bonds indicates the successful formation of an N‐doped carbon matrix. The N 1s XPS spectrum (Figure 2b) can be divided into five separate species, that is pyridinic N (398.7 eV), metal‐coordinated N (399.8 eV), graphitic N (400.9 eV), pyrrolic N (402.1 eV) and oxidized N (405.5 eV). The peak located at 399.8 eV provides further evidence for the binding of Fe and/or Ni species to nitrogen centers in FeNi‐NC@MWCNT.

To further identify the chemical state and coordination environment of the Fe and Ni sites in FeNi‐NC@MWCNT, X‐ray absorption near edge structure (XANES) and extended X‐ray absorption fine structure (EXAFS) analyses were conducted. As shown in Figure 2c, the Fe K‐edge XANES spectra reveal that the Fe energy absorption edge position of FeNi‐NC@MWCNT is located between Fe foil and Fe_2_O_3_, indicating the average oxidation state of Fe is between Fe(0) and Fe_2_O_3_ (+3). The inset graph in Figure 2c highlights the weak pre‐edge feature at ≈7115 eV, which is close to iron phthalocyanine (FePc) and can be assigned to the charge transfer from ligands to metal atoms and metal 1s to 3d transition in FeNi‐NC@MWCNT.^[^ ^39^ ^]^ Similarly, the XANES spectra of the Ni K‐edge for FeNi‐NC@MWCNT give an average valence of Ni between Ni(0) and Ni(+2). Note that the nickel XANES data show comparable features to nickel phthalocyanine (NiPc), suggesting the existence of a Ni‐N_x_ coordination environment in the FeNi‐NC@MWCNT (Figure 2d). Notably, the pre‐edge peak at ≈8335 eV of Ni curve in FeNi‐NC@MWCNT is higher than that of NiPc, implying a distorted D_4_ _h_ symmetry in Ni central atoms, which possibly results from Fe‐Ni interactions (Figure 2d insert). Figure 2e displays the Fourier‐transformed (FT) k^2^‐weighted Fe K‐edge EXAFS spectra in R‐space. Compared with the FePc reference, a pronounced shoulder of the Fe K‐edge at 1.4 Å indicates the Fe‐N_x_ coordination structure in FeNi‐NC@MWCNT. Notably, the Fe K‐edge curve of FeNi‐NC@MWCNT shows a major peak at 1.9 Å for Fe‐Ni scattering, which is different from the peak referred to Fe‐Fe interactions for Fe foil at 2.2 Å, indicating no iron metal nanoclusters in the catalyst. For the Ni K‐edge FT‐EXAFS, the main peak at 1.4 Å for FeNi‐NC@MWCNT is comparable to the peak in the NiPc curve representing the Ni‐N_x_ coordination structure, which is distinctive to the peaks at 1.6 Å for Ni‐O (NiO) and 2.2 Å for Ni‐Ni (Ni foil) (Figure 2f). Moreover, the peak at ≈1.8 Å for the Ni K‐edge EXAFS data of FeNi‐NC@MWCNT can be assigned to Ni‐Fe interactions. Besides, the formation of metal‐N_x_ (Fe‐N_x_, Ni‐N_x_) coordination in FeNi‐NC@MWCNT is further confirmed by Wavelet‐transform (WT)‐EXAFS spectra. Figure 2g presents the radial distance and k‐space resolution to recognize the Fe and Ni at the atomic level in the FeNi‐NC@MWCNT catalyst.

Compared with Fe foil, the location of the intensity maxima of FeNi‐NC@MWCNT shows left‐shift and down‐shift in the bond length R and wave vector k respectively, suggesting the Fe atoms are atomically dispersed into the catalyst in the form of Fe‐N coordination. The WT contour plot for FeNi‐NC@MWCNT also exhibits a maximum intensity at 6.5 Å^−1^ in k space, considerably lower that than of Ni foil (7.5 Å^−1^), indicating the atomic dispersion of Ni and the formation of Ni‐N bonds in FeNi‐NC@MWCNT. The XANES, FT‐EXAFS, and WT‐EXAFS results all suggest that the bulk of FeNi‐NC@MWCNT features Fe‐N_x_, Ni‐N_x,_ and FeNi‐N_x_ sites. Also, nonlinear least‐squares EXAFS fitting analysis was performed to quantitatively evaluate the local structural parameters for Fe species and Ni species in FeNi‐NC@MWCNT. As shown in Table S2 (Supporting Information), the coordination numbers of Fe‐N, Ni‐N, and Fe‐Ni in FeNi‐NC@MWCNT are estimated to be 2.6, 3.5, and 1.5 (average value) respectively, indicating the formation of Fe‐Ni interactions in FeNi‐NC@MWCNT. Furthermore, FT‐EXAFS k‐edge curves of Fe and Ni are both in agreement with the fitting curves simulated from the proposed structural model (Figures S9, S10, Supporting Information), suggesting FeNi‐N_6_ sites as a dominant feature in FeNi‐NC@MWCNT.

### 2.2 Electrocatalytic ORR and OER Performance

The bifunctional ORR/OER performance of the native FeNi‐NC@MWCNT catalyst was investigated with a half‐cell setup based on a typical three‐electrode configuration (see Supporting Information for details). All potentials reported were referenced to reversible hydrogen electrode potentials (RHE). The performance of the reference materials Fe‐NC@MWCNTs, Ni‐NC@MWCNTs, FeNi‐NC, and commercial Pt/C, IrO_2_ were also evaluated for comparison.

**Figure 3:**
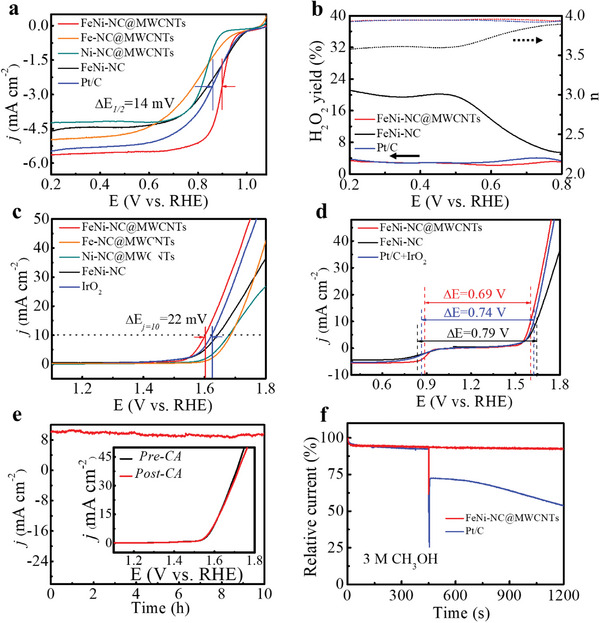
ORR/OER activity and stability of as‐synthesized catalysts in 0.1 m KOH solution. a) ORR polarization curves at 1600 rpm of FeNi‐NC@MWCNT, Fe‐NC@MWCNTs, Ni‐NC@MWCNTs, FeNi‐NC, and commercial Pt/C. b) H_2_O_2_ yield and electron transfer numbers of FeNi‐NC@MWCNT, FeNi‐NC, and Pt/C. c) OER polarization curves of studied catalysts. d) Overall polarization curves of FeNi‐NC@MWCNT, FeNi‐NC, and Pt/C+IrO_2_ for bifunctional catalytic activity tests. e) Chronoamperometry curves of FeNi‐NC@MWCNT at 1.65 V (vs RHE) without iR compensation (insert: polarization curves comparison before and after 10 h stability test). f) Relative i‐t curves of FeNi‐NC@MWCNT and Pt/C at 0.6 V (vs RHE) and 1600 rpm with 3 m methanol was added at ca. 450 s.

The ORR tests were conducted in an O_2_‐saturated 0.1 M aqueous KOH solution using a rotating disk electrode (RDE) as a working electrode. Compared with the negligible background currents in an Ar‐saturated solution, the cyclic voltammetry (CV) of FeNi‐NC@MWCNT in an O_2_‐saturated solution shows a major cathodic oxygen reduction peak at 0.77 V (Figure S11, Supporting Information). As shown in the linear sweep voltammetry (LSV) curves (**Figure** **3**a), FeNi‐NC@MWCNT exhibits superior ORR catalytic activity in terms of high onset potential (0.99 V), high half‐wave potential (E_1/2_) (0.9 V) and large limiting current density (5.63 mA cm^−2^), which surpass these values of the references Fe‐NC@MWCNTs (0.91 V, 0.88 V, 4.86 mA cm^−2^), Ni‐NC@MWCNTs (0.89 V, 0.83 V, 4.18 mA cm^−2^), FeNi‐NC (0.93 V, 0.87 V, 4.43 mA cm^−2^), and even outperforms that of commercial Pt/C (0.95 V, 0.88 V, 5.45 mA cm^−2^). In fact, the performance of FeNi‐NC@MWCNT is superior to most currently reported SAC or DAC materials based on non‐precious metals (Table S5, Supporting Information). Comparison of the title compound with the single‐metal species Fe‐NC@MWCNTs and Ni‐NC@MWCNTs as well as the MWCNT‐free sample FeNi‐NC demonstrates the impact of the dual metal sites and the carbon nanotube linkage. FeNi‐NC@MWCNT also exhibits a smaller Tafel slope (48.5 mV dec^−1^) than FeNi‐NC (62.8 mV dec^−1^) and Pt/C (58.4 mV dec^−1^), indicating a faster ORR electrocatalytic kinetics (Figure S12, Supporting Information). Furthermore, the electron transfer number (*n*) and selectivity of FeNi‐NC@MWCNT as well as its counterparts were evaluated by rotating ring disk electrode (RRDE) measurements. As shown in Figure 3b, the peroxide yield of FeNi‐NC@MWCNT is below 3.1% and the monitored *n* is above 3.9 over the potential range of 0.2–0.8 V, verifying the high ORR selectivity and dominant 4 e^−^ transfer mechanism. In contrast, the FeNi‐NC shows a much higher peroxide yield (20%) and lower *n* value of 3.6 at 0.48 V versus RHE, suggesting an enhanced activity retention of FeNi‐NC@MWCNT. The average *n* value of FeNi‐NC@MWCNT was also calculated to be ≈3.98 (Figure S13, Supporting Information) using Koutecky‐Levich (K‐L) analysis of polarization curves obtained at different electrode rotation rates, further confirming a 4e^−^ transfer pathway. Moreover, the K‐L plots for FeNi‐NC@MWCNT at different potentials exhibit parallel linear correlation (Figure S13, Supporting Information), indicating its potential‐independent electron transfer rate and the first‐order reaction kinetics with O_2_ concentration.

To explore the reactivity of our catalyst as bifunctional ORR/OER electrocatalysis in rechargeable Zn–air batteries, we explored both reactivities in detail. First, the OER performance of the catalysts was investigated in 0.1 m aqueous KOH. As shown in Figure 3c, FeNi‐NC@MWCNT shows an OER overpotential of 370 mV at j = 10 mA cm^−2^, which is significantly lower than Fe‐NC@MWCNT (473 mV), Ni‐NC@MWCNT (454 mV), FeNi‐NC (417 mV) and even commercial IrO_2_ (390 mV). In addition, FeNi‐NC@MWCNT also shows the lowest Tafel slope (63.5 mV dec^−1^) amongst the materials tested (Figure S12b, Supporting Information). The composite also outperforms many other recently reported OER catalysts (Table S5, Supporting Information).

Electrochemical impedance spectroscopy (EIS) was performed at a potential of 1.56 V to further evaluate the OER kinetics. The diameter of the semicircle of the EIS spectrum for FeNi‐NC@MWCNT is much smaller than that of the reference catalysts, revealing more efficient charge transfer and lower overall impedance, leading to faster OER kinetics (Figure S14a, Supporting Information). The potential gap ∆E (the difference between the half‐wave potential (E_1/2_) of ORR and the OER potential at 10 mA cm^−2^ (E_j = 10_)) is used as an indicator to evaluate the bifunctional activity of the studied oxygen catalysts. Note that smaller ∆E values imply high bifunctional OER/ORR performance. Figure 3d shows that FeNi‐NC@MWCNT has a ∆E value of 0.69 V, which is lower than that of FeNi‐NC (0.79 V) and Pt/C+IrO_2_ (0.74 V). The above analyses collectively demonstrate the high ORR and OER catalytic activity of FeNi‐NC@MWCNT.

Durability is another key performance indicator for the practical applicability of bifunctional oxygen catalysts, e.g., in rechargeable ZABs. Thus, the stability of the catalysts for both ORR and OER was assessed by chronoamperometry (CA). As shown in Figure S14b (Supporting Information), after 24 h of CA for ORR at a constant potential of 0.8 V in O_2_‐saturated electrolyte, FeNi‐NC@MWCNT retained 93.5% of the initial current density, while significantly higher current density drops were observed for the references FeNi‐NC (81.5%) and Pt/C (52.6%). The superior durability of FeNi‐NC@MWCNT is further corroborated by virtually identical LSV curves before and after the CA analysis (Figure S15a, Supporting Information).

Furthermore, its robustness was further shown by negligible loss of the chronoamperometric OER performance over 10 h polarization at 1.62 V (Figure 3e), which is further confirmed by the essentially identical LSV curves before and after CA (Insert in Figure 3e). Furthermore, the OER faradaic efficiency of FeNi‐NC@MWCNT was also assessed with RRDE voltammetry. In this study, the ring potential was set as E = 0.40 V to reductively detect the oxygen generated at the disk electrode. According to the monitored ring current, disk current, and current collection efficiency (0.15), the OER faradaic efficiency of FeNi‐NC@MWCNT was determined as 95.6%, which is higher than that of the reference catalysts (Figure S15b, Supporting Information). The electrochemically active surface area (ECSA) was further investigated to assess the number of accessible reactive sites, which is correlated with double‐layer capacitance (*C*_dl_). As shown in Figures S12c, S18 (Supporting Information), the *C*_dl_ of FeNi‐NC@MWCNT (18.81 mF cm^−2^) is considerably larger than that of FeNi‐NC (12.35 mF cm^−2^) and Pt/C (6.14 mF cm^−2^), which also maintained a value of 17.96 mF cm^−2^ post OER catalysis.

Considering the high ORR activity of FeNi‐NC@MWCNT, it could be used as a potential cathodic catalyst in direct‐methanol fuel cells. In order to estimate the methanol tolerance of the catalysts, CA analysis for ORR at 0.8 V was studied by RDE at 1600 rpm in the presence of 3 m methanol in 0.1 m aqueous KOH electrolyte. As illustrated in Figure 3f, the relative current density of FeNi‐NC@MWCNT shows minimal change over the measurement, indicating the high methanol tolerance. In sharp contrast, the current density of the Pt/C catalyst drops rapidly after injecting methanol under identical conditions.

Moreover, to reveal the structural evolution of the FeNi‐NC@MWCNTs during the ORR/OER catalytic process, we collected the catalysts after a chronoamperometric test and performed HAADF‐STEM measurements. These initial analyses suggest a partial loss and/or aggregation of the metal species after ORR or OER catalysis (Figures S16, S17). However, note that these structural changes did not affect the chronoamperometric performance, see above.

### 2.3 Primary and Rechargeable Zinc–Air Batteries Test

**Figure 4:**
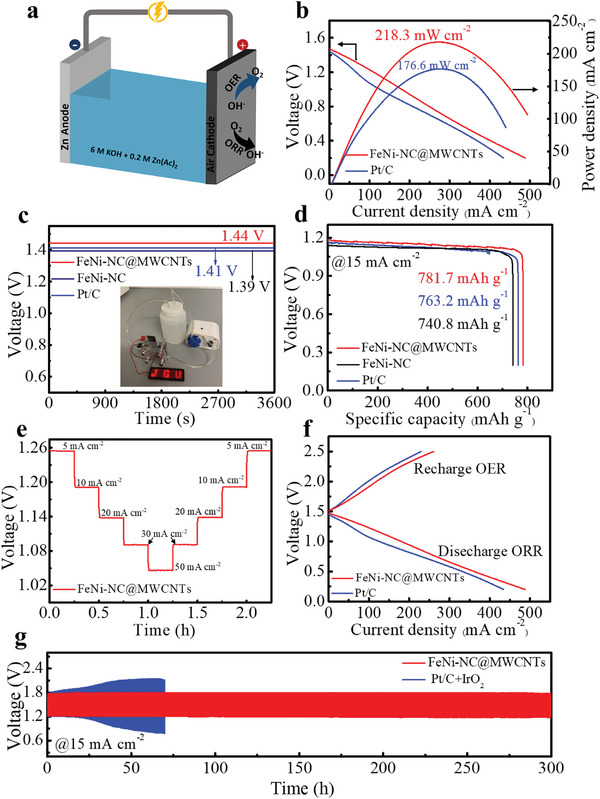
Zn–air batteries (ZAB) performance. a) Schematic illustration of the ZAB setup. b) Discharge polarization curves and corresponding power density curves of FeNi‐NC@MWNTs and Pt/C based ZABs. c) Open‐circuit plots of FeNi‐NC@MWCNT, FeNi‐NC, and Pt/C based ZABs. (insert: ZAB powers the LED screen). d) Discharge plots of primary ZABs catalyzed by FeNi‐NC@MWCNT, FeNi‐NC, and Pt/C at a current density of 15 mA cm^−2^. e) Discharge curves of FeNi‐NC@MWCNT‐based ZABs at different current densities of 5, 10, 20, 30, and 50 mA cm^−2^. f) Polarization curves of FeNi‐NC@MWCNT and Pt/C+IrO_2_ catalyzed ZABs. g) Galvanostatic charge‐discharge cycling performance of ZABs based on FeNi‐NC@MWCNT and Pt/C+IrO_2_ at a current density of 15 mA cm^−2^.

Considering the outstanding bifunctional catalytic activity of FeNi‐NC@MWCNT, its practical performances in primary and rechargeable Zn–air batteries have been evaluated with custom‐designed cells. As schematically illustrated in **Figure** **4**a, a customized two‐electrode ZAB was assembled utilizing an air cathode loaded with FeNi‐NC@MWCNT as a catalyst. A zinc metal plate was used as anode. The electrolyte was 6.0 m aqueous KOH containing 0.2 m Zn(OAc)_2_. The electrolyte was continuously cycled by a peristaltic pump to prevent local concentration gradients. As references, ZABs using FeNi‐NC and a mixture of commercial Pt/C + IrO_2_ (with a mass ratio of 1:1) as cathode catalyst were fabricated. As shown in Figure 4b, the discharge polarization curves (at *j* = 260 mA cm^−2^) show that the ZAB catalyzed by FeNi‐NC@MWCNT exhibits a maximum power density of 218.3 mW cm^−2^, which is considerably higher than for Pt/C + IrO_2_ (176.6 mW cm^−2^). The ZAB based on FeNi‐NC@MWCNT also delivers a stable open‐circuit voltage (OCV) of 1.44 V with no obvious changes over 1 h (Figure 4c), which is marginally higher than for FeNi‐NC (1.41 V) and Pt/C + IrO_2_ (1.39 V). These observations are in line with the catalytic activities in the three‐electrode configuration reported above. The inserted picture in Figure 4c shows the LED display (1–5 V) was successfully lit up with the FeNi‐NC@MWCNT‐based ZAB, demonstrating the practical application of this catalyst. As shown in Figure 4d, the FeNi‐NC@MWCNT containing ZAB shows a specific capacity of 781.7 mAh g^−1^ (normalized to the mass of consumed Zn), which outperforms the capacities of a Fe‐NC‐based ZAB (740.8 mAh g^−1^) and Pt/C based ZAB (763.2 mAh g^−1^). The output voltage of the FeNi‐NC@MWCNT ZAB is approx. 1.18 V, which is superior to FeNi‐NC (1.13 V) and Pt/C (1.16 V). Furthermore, as demonstrated in Figure S19, the primary ZAB with FeNi‐NC@MWCNT catalyst was discharged under galvanostatic conditions (15 mA cm^−2^) for more than 21 h without a noticeable voltage drop. Figure 4e shows the remarkable discharging rate capacity and durability of FeNi‐NC@MWCNT‐based ZAB at various discharge current densities ranging from 5 to 50 mA cm^−2^, no significant voltage loss was observed with increasing discharge current. Notably, the initial and final discharge voltage of the FeNi‐NC@MWCNT‐based ZAB at 1 mA cm^−2^ remains unchanged (1.25 V), suggesting a robust long‐term discharge capacity.

To further investigate the rechargeability of the ZABs, the long‐term cycling stability of the ZABs catalyzed by FeNi‐NC@MWCNT was tested by continuous galvanostatic charge/discharge at a current density of 15 mA cm^−2^. The duration of each cycle was set to 1 h (0.5 h for discharging and 0.5 h for subsequent charging) with an initial charge voltage of 1.8 V and discharge voltage of 1.15 V, respectively. The ZABs with commercial Pt/C +IrO_2_ were examined under identical conditions for comparison. As shown in Figure 4f, the ZAB assembled with FeNi‐NC@MWCNT exhibits a higher discharge current density and a lower charge/discharge voltage gap compared to the ZABs based on Pt/C+ IrO_2_. In addition, the round‐trip efficiency of the ZABs was calculated based on the charge/discharge voltage gap of the second cycle. The ZAB with FeNi‐NC@MWCNT exhibits a high round‐trip efficiency of 73% (Figure S20, Supporting Information), surpassing that of the Pt/C + IrO_2_ catalyzed ZAB (65%). For the long‐term cycling test, as shown in Figure 4g, the FeNi‐NC@MWCNT catalyzed ZAB demonstrates a narrower charge/discharge voltage gap and negligible voltage drop after 300 h cycling, suggesting its superior durability which outperforms most of the recently reported SAC/DAC catalyzed ZABs (Table S6, Supporting Information). In contrast, a substantial charge‐discharge voltage drop was observed in the reference ZAB with Pt/C**+**IrO_2_ mixture after 75 h. Based on the above results, the FeNi‐NC@MWCNT is a promising and robust alternative to precious metal‐based catalysts for both primary and rechargeable Zn–air batteries.

### 2.4 Density Functional Theory Calculation

To further elucidate the catalytic activity and reaction mechanism of FeNi DACs for ORR and OER, first‐principles calculations were performed using density functional theory (DFT) methods. Different types of models for both heteronuclear and homonuclear sites FeNiN_6_, FeN_4_, NiN_4_ were constructed with coordination environments based on experimental XANES/EXAFS results (Figure S21, Supporting Information). Also, a model for nonadjacent FeN_4_‐NiN_4_ sites was constructed as a reference to assess the critical role of adjacent hetero‐bimetallic active sites. According to the adsorption energy of OH* (ΔG_OH*_) on active sites in different models (Table S3, Supporting Information), the pristine FeNiN_6_ site exhibits a negative adsorption energy of ‐0.24 V for OH*, suggesting the strong adsorption and difficult desorption of OH* on the metal sites. Thus, the OH* would be spontaneously adsorbed on the metal sites during catalysis in alkaline media, and it has been reported that OH* serves as a modifying ligand to optimize the electronic configuration of metal sites and influence their catalytic ORR/OER activity.^[^ ^40^ ^]^ As depicted in Figure S22 (Supporting Information), the FeNi‐NC model is the favorable configuration with OH* pre‐adsorbed on the Fe site of FeNiN_6_, denoted as FeNi‐OH‐NC.

Based on EXAFS fitting, we proposed that FeNiN_6_ (FeNi‐OH‐NC) could be the main contributor to the enhanced catalytic performance of FeNi‐NC@MWCNT. Therefore, the FeNi‐OH‐NC configuration was chosen as the key model for the subsequent ORR/OER computational investigations, and Fe‐NC, Ni‐NC, and nonadjacent Fe‐Ni‐NC were also simulated for comparative investigation. Particularly considering the possible reactive sites for the nonadjacent Fe‐Ni‐NC, two models of Fe@Fe‐Ni‐NC (on the Fe site) and Ni@Fe‐Ni‐NC (on the Ni site) were both calculated for the ORR/OER. Note that the optimized results for the adjacent FeNi‐OH‐NC model suggest that Fe is the reactive site.

**Figure 5:**
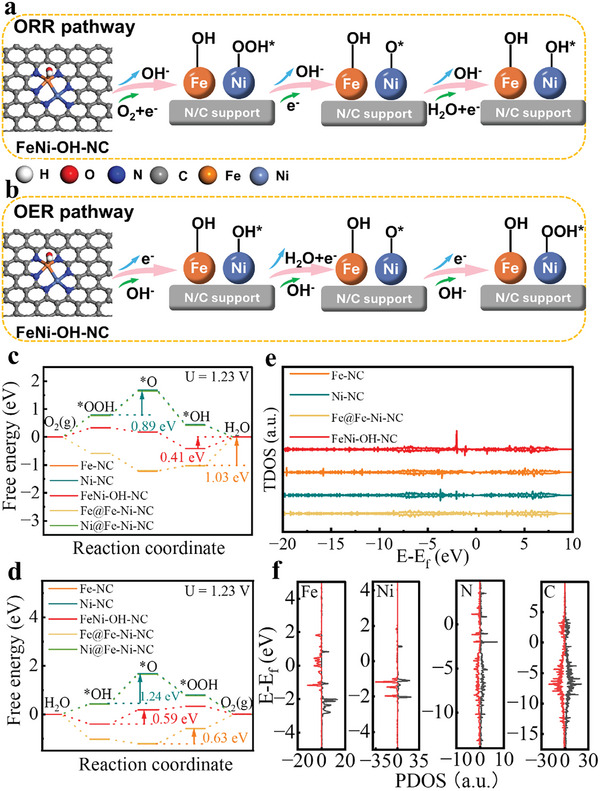
Density functional theory (DFT) calculations. a) Proposed mechanism for ORR and b) for OER on FeNi‐OH‐NC model. The grey, blue, orange, and cyan balls represent carbon, nitrogen, iron, and nickel atoms, respectively. c) Free energy diagrams of ORR intermediates on Fe‐NC, Ni‐NC, Fe@Fe‐Ni‐NC, Ni@Fe‐Ni‐NC, and FeNi‐OH‐NC models at different potentials. d) Free energy diagrams of OER intermediates on Fe‐NC, Ni‐NC, Fe@Fe‐Ni‐NC, Ni@Fe‐Ni‐NC, and FeNi‐OH‐NC models at different potentials. e) The total density of states (TDOS) of Fe‐NC, Ni‐NC, Fe@Fe‐Ni‐NC, Ni@Fe‐Ni‐NC, and FeNi‐OH‐NC models. f) Projected density of states (PDOS) of Fe, Ni, N, and C for FeNi‐OH‐NC model.

**Figure** **5**a illustrates the proposed 4e^−a^ pathway for the ORR mechanism on optimized FeNiN_6_ sites in the FeNi‐OH‐NC model (other possible models and their oxygen‐containing intermediates are depicted in Figures S23–S28, Supporting Information). The corresponding ORR calculated Gibbs free energy diagrams of these constructed models are evaluated at different electrode potentials (Figure S29, Supporting Information). At the potential U of 0 V, the four elementary steps are energetically downhill for all models, indicating all the involved reaction steps are exothermic in nature. When the potential is at 0.86 V, the fourth step (OH* + OH^−^ → O* + H_2_O + e^−^) is recognized as the rate‐determining step (RDS) for the FeNi‐OH‐NC model with a free energy difference of only 0.04 eV.

In comparison, the RDS for both Fe@Fe‐Ni‐NC and Fe‐NC is also the fourth step with corresponding variation in free energy of 0.67 and 0.66 eV, respectively. The RDS for Ni‐NC and Ni@Fe‐Ni‐NC is the second step (OOH* + e^−^ → O* + OH^−^) with corresponding variation in free energy of 0.52 and 0.51 eV, respectively. When the potential U increases up to 1.23 V, the fourth step is still recognized as the RDS for the FeNi‐OH‐NC model and the calculated free energy difference is determined as 0.41 eV, which is lower than those of Fe@Fe‐Ni‐NC (1.04 eV) and Fe‐NC (1.03 eV) with the same RDS, and also lower than those of Ni‐NC (0.89 eV) and Ni@Fe‐Ni‐NC (0.88 eV) with RDS in the second step, suggesting the FeNi‐OH‐NC has the lowest energy barrier for ORR. Notably, the free energies of each step for the Fe‐NC and Fe@Fe‐Ni‐NC model are close to each other at the three investigated potentials, as are those for the Ni‐NC and Ni@Fe‐Ni‐NC models, indicating that the nonadjacent metal sites can't leverage the synergistic effects. Figure 5b displays the proposed reaction mechanism for the OER process, the corresponding OER free energy diagrams of these constructed models are shown in Figure 5d and Figure S29c,d (Supporting Information). At the potential of 0 V (Figure S29c, Supporting Information), all the interaction steps are uphill, implying that the OER is energetically unfavorable at this potential. When U = 1.23 V, the RDS of FeNi‐OH‐NC is the second step (OH* + OH^−^ → O* + H_2_O + e^−^) with a free energy difference of 0.59 eV, which is the lowest compared with those of the other four models as shown in Figure 5d. When U = 1.57 V (Figure S29d), the free energy difference for the RDS of FeNi‐OH‐NC towardsOER is evaluated as 0.25 eV, which is also lower than those of the other four models. Similarly, the Fe‐NC model exhibits comparably overlapped free energies diagrams with the nonadjacent Fe@Fe‐Ni‐NC model, as are those for the Ni‐NC and Ni@Fe‐Ni‐NC models. All the above results indicate that the adjacent FeNi heteroatom configuration as an active site effectively promote ORR/OER catalytic performance.

The ORR/OER catalytic activity can also be evaluated by considering the adsorption energy (∆G_OH*_) and ∆G_*O_ – ∆G_*OH_ as indicators to establish a volcano‐shaped curve with ORR/OER overpotentials. Figure S30a (Supporting Information) shows the ORR overpotential *η* versus ∆G_OH*_, the FeNi‐OH‐NC model is located at the top of the volcano curve with the smallest overpotential of 0.41 V, which is significantly lower than the monometallic models Fe‐NC (1.03 V), Ni‐NC (0.89 V) and the nonadjacent bimetallic models Fe@Fe‐Ni‐NC (1.04 V) and Ni@Fe‐Ni‐NC (0.88 V). Thus, the theoretically calculated trends of the ORR reactivity are consistent with the experimental results. Similarly, FeNi‐OH‐NC also exhibits the best OER catalytic activity with an overpotential of 0.59 V (Figure S30b, Supporting Information), followed by Fe@Fe‐Ni‐NC (0.61 V), Fe‐NC (0.63 V), Ni@Fe‐Ni‐NC (1.23 V) and Ni‐NC (1.24 V). Here also, theory supports the experimental findings.

Furthermore, as shown in Figure S31 (Supporting Information), the charge distribution analysis reveals that N atoms in the FeNi‐OH‐NC model are more negatively charged compared to Fe‐NC, Ni‐NC, and nonadjacent Fe@Fe‐Ni‐NC models, suggesting the formation of metal‐metal interactions of adjacent Fe‐Ni sites in FeNi‐OH‐NC. This feature is expected to enhance the charge transfer and electron redistribution at the active sites. This modifies the electronic configuration of the Fe and Ni atoms, and facilitates the adsorption of oxygen intermediates, resulting in improved catalytic activity.

Furthermore, analysis of the total density of states (TDOS) shown in Figure 5e suggests that FeNi‐OH‐NC features a higher density of states across the Fermi level (followed by Ni‐NC, Fe@Fe‐Ni‐NC, and Fe‐NC), indicating the highest electronic conductivity of the FeNi‐OH‐NC model. This calculated finding is in agreement with the EIS experimental results described above. Figure 5f presents the detailed projected density of states (PDOS) for Fe 3d, Ni 3d, N 2p, and C 2p orbitals of the FeNi‐OH‐NC model. Compared with monometallic models Fe‐NC, Ni‐NC and nonadjacent bimetallic Fe@Fe‐Ni‐NC models (Figures S32–S34, Supporting Information), the adjacent bimetallic FeNi‐OH‐NC model demonstrates more hybrid electrons near the Fermi level, implying the existence of electronic interaction of Fe, Ni, and N on the active sites. Therefore, the DFT analysis demonstrates the adjacent diatomic FeNi‐OH‐NC configuration induces subtle modulation of the asymmetric electronic configuration of FeNiN_6_ active sites, which facilitates the kinetics of the electrocatalytic ORR/OER process.

## 3 Conclusion

A dual‐atom catalyst based on adjacent iron and nickel sites is reported. The catalyst shows outstanding performance for both ORR and OER, which allows the integration into high‐performance rechargeable Zinc–air batteries that use the new bifunctional catalyst for both ORR and OER. Experimental and theoretical analyses rationalize the observed catalytic performance and robustness. Mechanistic studies into the nature of the catalytic site allow an initial understanding of the role and synergism of the metal centers as well as the N‐doped carbon matrix and the conductive carbon nanotube support structure. The results suggest that OH* is pre‐adsorbed on the Fe site of FeNiN_6_, while the adjacent diatomic FeNi‐OH‐NC configuration induces subtle modulation of the asymmetric electronic configuration of FeNiN_6_ active sites. The work provides an effective strategy to develop advanced bifunctional catalyst for critical energy conversion and storage processes.

## Conflict of Interest

The authors declare no conflict of interest.

## Supporting information



Supporting Information

## Data Availability

The data that support the findings of this study are openly available in Zenodo.org at DOI 10.5281/zenodo.13336766, reference number 13336766.
