# Supplementary material for: A Bifunctional Iron‐Nickel Oxygen Reduction/Oxygen Evolution Catalyst for High‐Performance Rechargeable Zinc–Air Batteries
Source: Small. 2024 Nov 27;21(3):2409161. doi: 10.1002/smll.202409161 (PMC11753497; doi:10.1002/smll.202409161)
Supplement: Supplementary file 1 — Supporting Information [file SMLL-21-2409161-s001.pdf]

NANO · MICRO
small

Supporting Information

for *Small*, DOI 10.1002/smll.202409161

# A Bifunctional Iron-Nickel Oxygen Reduction/Oxygen Evolution Catalyst for High-Performance Rechargeable Zinc–Air Batteries

*Zhengfan Chen, Weiyi Cheng, Kecheng Cao, Meng Jin, Sarra Rahali, Soressa Abera Chala, Elnaz Ebrahimi, Nana Ma*, Rongji Liu*, Keseven Lakshmanan, Chia-Yu Chang, Chun-Chi Cheung, Haojian Luo, Yongkang Wang, Bing Joe Hwang* and Carsten Streb**

# Supporting Information

## A Bifunctional Iron-Nickel Oxygen Reduction / Oxygen Evolution Catalyst for High-Performance Rechargeable Zinc-Air Batteries

Zhengfan Chen,[a] Weiyi Cheng,[b] Kecheng Cao,[d] Meng Jin,[d] Sarra Rahali,[a] Soressa Abera Chala,[a] Elnaz Ebrahimi,[a] Nana Ma*[b], Rongji Liu*[a], Keseven Lakshmanan,[c] Chia-Yu Chang,[c] Chun-Chi Cheung,[c] Haojian Luo,[e] Yongkang Wang,[e] Bing Joe Hwang*[c], Carsten Streb*[a]

[a] M.Sc. Z. Chen, Dr. S. Rahali, Dr. S. A. Chala, M.Sc. E. Ebrahimi, Dr. R. Liu, Prof. Dr. C. Streb
Department of Chemistry
Johannes Gutenberg University Mainz
Duesbergweg 10-14, 55128 Mainz, (Germany)
E-mail: rongji.liu@uni-mainz.de, carsten.streb@uni-mainz.de.

[b] Dr. N. Ma, M.Sc. W. Cheng
School of Chemistry and Chemical Engineering
Henan Normal University
453007 Xinxiang (China)
E-mail: mann076@htu.edu.cn

[c] Prof. Dr. B. Hwang, Dr. K. Lakshmanan, M.Sc. C, Chang, M.Sc. C, Cheung
Sustainable Electrochemical Energy Development (SEED) Center
National Taiwan University of Science and Technology
106335 Taipei (Taiwan)
E-mail: bjh@mail.ntust.edu.tw

[d] Dr. K. Cao, M.Sc. M. Jin
School of Physical Science and Technology
Shanghai Tech University
201210 Shanghai (China)

[e] M.Sc. H. Luo, Dr. Y. Wang
Department of Molecular Spectroscopy
Max Planck Institute for Polymer Research
55128 Mainz (Germany)

## I. Experimental procedures

**Materials and general information for synthesis:** All reagents and solvents were purchased from commercial suppliers and were used without further purification.

**Synthesis of FeNi-NC@MWCNTs:** 2-methylimidazole (1.68 g, 20 mmol) and 60 mg multi-walled carbon nanotubes (MWCNTs) were dispersed in 40 ml methanol with stirring, then a solution containing methanol (30 ml), $Zn(NO_3)_2{\cdot}6H_2O$ (1.19 g, 4 mmol), $Fe(acac)_3$ (70.63 mg, 0.2 mmol) and $Ni(NO_3)_2{\cdot}6H_2O$ (58.2 mg, 0.2 mmol) was added dropwise into the above solution and stirred vigorously for 6 h at room temperature. The resulting precipitate was collected by centrifugation and washed with methanol, then dried in a vacuum oven at 50 °C overnight. The resulting powder was transferred into ceramic boat and was pyrolyzed in a tube furnace under an Ar flow by elevating the temperature from room temperature to 950 °C at a rate of 5 °C $min^{-1}$, maintaining at 950 °C for 3 h and then cooling down naturally. The pyrolysis products were etched with 1.5 M aqueous HCl (ca. 15 ml) to remove

Zn and ZnO (formed during the carbonation process), then final products were obtained after rinsing with DI water and drying.

**Synthesis of Fe-NC@MWCNTs:** Fe-NC@MWCNTs was obtained with the same procedure as above, without adding $Ni(NO_3)_2·6H_2O$ in the procedure.

**Synthesis of Ni-NC@MWCNTs:** Ni-NC@MWCNTs was obtained with the same procedure as above, without adding $Fe(acac)_3$ in the procedure.

**Synthesis of FeNi-NC:** FeNi-NC was obtained with the same procedure as above, without adding MWCNTs in the procedure.

**Powder X-ray diffraction** was measured on Bruker D2 Phaser equipment with Cu Kα radiation (λ = 1.5406 Å) at a scan rate of 15 °/min in the 2θ range from 5° to 90°.

**Raman spectroscopy** was recorded with a WITec confocal Raman spectrometer (Alpha 300 R, ×50 objective) with 600 grooves/mm grating, and 532 nm excitation laser at 0.25 mW power.

**X-ray photoelectron spectroscopy** (XPS) was performed using a PHI Quantera SXM system with monochromatized Al Kα radiation.

**Scanning electron microscopy** (SEM) images were obtained with Nova NanoSEM at 15 kV acceleration voltage.

**Aberration-corrected high-angle annular dark-field scanning TEM** (AC-HAADF-STEM) images and the elemental mapping images were acquired on a JEM-ARM300F.

**Inductively coupled plasma optical emission spectroscopy** (ICP OES) was performed using an Agilent 5800 VDV optical emission spectrometer with an automatic sampler SPS 4.

**X-ray absorption spectroscopy** (XAS) was obtained on the hard X-ray spectroscopy beamline in the Taiwan Synchrotron Radiation Facility.

**Electrochemical measurements:** electrochemical analyses of the as-synthesized catalysts were investigated with an electrochemical workstation (CHI 760E) with a standard three-electrode electrochemical cell. A saturated calomel electrode (SCE) and graphite rod were used as reference electrode and counter electrode, respectively. A glassy carbon rotating disk electrode (RDE with a glassy carbon surface area of 0.1256 $cm^2$) and a rotating Pt ring-glassy carbon disk electrode (RRDE with a glassy carbon surface area of 0.1256 $cm^2$ and a Pt ring surface area of 0.1884 $cm^2$) were used as working electrodes to evaluate electrochemical catalytic performance. All electrode potentials in this study were converted to the reversible hydrogen electrode (RHE) with the following equation:

$$E_{RHE} = E_{SCE} + 0.241 + 0.059 \times pH \quad (1)$$

The catalyst inks were prepared by the following method, 5 mg as-synthesized catalyst, 20 μL $H_2O$ and 20 μL Nafion solution (5 wt% in water and 1-propanol, Nafion D-520, Germany) were dispersed in 960 μL ethanol to form a homogeneous ink by ultrasonication for 1 h. Then 7.54 μL of the catalyst ink was uniformly drop-cast on the surface of the glassy carbon working electrode and air-dried at room temperature, the mass loading of the catalyst is approx. 0.3 mg $cm^{-2}$. Before catalyst loading, the RDE or RRDE was mechanically polished with $Al_2O_3$ slurry to obtain a mirror-like surface.

Before the tests, the 0.1 M aqueous KOH electrolyte was purged with oxygen or argon constantly for at least 30 min, ensuring that the electrolyte was in $O_2$-saturated or Ar-saturated state. Cyclic voltammetry (CV) measurements were carried out at a scan rate of 100 mV $s^{-1}$, and the linear sweep voltammetry (LSV) curves were performed at a scan rate of 5 mV $s^{-1}$ with 90% IR compensation to evaluate the catalytic activity. For the ORR/OER catalytic activity evaluation, the ORR polarization curves were recorded from 0 V to 1.25 V (vs. RHE), and from 1.01 V to1.7 V (vs. RHE) for OER with a rotation rate range from 400 to 1600 rpm. The durability of the catalysts was measured by chronoamperometry at $E_{10}$ (potential at j = 10 mA $cm^{-2}$) for OER and $E_{1/2}$ (half-wave potential) for ORR.

The Tafel slopes were calculated by the following formula:

$$\eta = a + b \times \log j \quad (2)$$

in Where $\eta$ is the corresponding overpotential at a specific current density, *a* is the Tafel constant, *b* is the Tafel slope, and *j* is the current density.

The electron transfer number and the hydrogen peroxide yield ($H_2O_2$ %) were calculated to evaluate the selectivity of the catalysts for oxygen reduction. The electrolyte was saturated with oxygen before the test. In this work, the potential of Pt ring on the RRDE was set as a constant value of 1.4 V (vs. RHE) and the LSV curves were recorded with RRDE rotating rate of 1600 rpm. The hydrogen peroxide yield $H_2O_2$% and the average electron transfer number *n* were calculated using the equations as below:

$$H_2O_2\% = 200 \times \frac{Ir/N}{Id+Ir/N} \quad (3)$$

$$n = 4 \times \frac{Id}{Id+Ir/N} \quad (4)$$

$I_r$ : ring current, $I_d$ : disk current, $N$ = 0.37 (current collection efficiency of the RRDE system)

Faradaic efficiency for OER was also calculated based on RRDE experiments. The electrolyte was saturated with Argon before the test. In this study, a constant potential (1.58 V vs. RHE) was applied at the disk electrode, while the potential of ring electrode was set as 0.4 V vs. RHE to reduce the $O_2$ produced from the disk. The Faradaic efficiency (FE) was obtained using the following equation:

$$FE=Ir/(Id\times N) \quad (5)$$

where $I_r$ and $I_d$ are the ring current and disk current, respectively. Here, $N$ is the current collection efficiency of the RRDE, which is determined as 15.7% in this system.

The electrochemically active surface area (ECSA) of all materials was determined via the electrochemical double-layer ($C_{dl}$) capacitance using the below equation,

$$C_{dl} = 4 \times \frac{Ic}{v} \quad (6)$$

The $C_{dl}$ was obtained from the CVs at different scan rates in a small potential range of 1.06 to 1.16 V vs. RHE, where no redox processes occur. $I_c$ is the charging current (mA $cm^{-2}$), $v$ is the scan rate (mV $s^{-1}$). There is a linear relationship between the current and scan rate, and the slope of the plot was used to calculate $C_{dl}$.

Electrochemical impedance spectroscopy (EIS) of the as prepared catalysts were measured at a constant potential of 1.55 V vs. RHE with an AC amplitude of 5 mV by sweeping the frequency from 100 kHz to ~0.01 Hz.

**Construction and test of zinc-air battery test:** The performance of these bifunctional catalyst in zinc-air batteries (ZABs) was investigated with a custom-built cell. A zinc plate with thickness of 0.5 mm was used as anode, which was polished with commercial sandpaper to remove the surface oxide layer. The air cathode was designed as a sandwich structure which is comprised of nickel foam, gas diffusion layer and catalytic layer (commercially obtained from Changsha Spring New Energy Technology Co., Ltd). Notably, the gas diffusion layer is waterproof but breathable. The catalyst ink was prepared by adding 15 mg synthesized catalyst (for instance, FeNi-NC@MWCNTs) in a solution of 1.9 ml ethanol, 50 μL $H_2O$ and 50 μL Nafion. After sonication for 1 h, the catalyst ink was loaded uniformly on the air cathode with a loading amount of 1.5 mg $cm^{-2}$. The prepared air cathode was dried at 25 °C for 24 hours to remove excess solvent. The electrolyte was a mixture solution of 6 M KOH and 0.2 M zinc acetate, the electrolyte was circulated through the battery by a peristaltic pump continuously to prevent the buildup of local concentrations of reagents. For comparison, zinc-air batteries with FeNi-NC and commercial catalyst Pt/C +$IrO_2$ (1:1 by mass ratio) as cathode catalyst were also assembled. The preparation of FeNi-NC and Pt/C-$IrO_2$ catalyst layers were performed following the above procedure.

All ZABs measurements were performed with a multi-channel battery testing system (Neware, CT-4008T5V6A-S1) at room temperature under ambient conditions. The power density of ZABs was determined by the polarization curves, the specific capacity was measured by a constant discharge current of 15 mA $cm^{-2}$ then normalized to the mass loss of the Zn anode. The rate capability of ZABs was evaluated at various current densities of 5, 10, 20, 30 and 50 mA $cm^{-2}$, respectively. The ZAB cycling durability was tested by charging and discharging at a constant current density of 15 mA $cm^{-2}$ (each cycle lasted 60 min, 30 min for charging and 30 min for discharging).

For the general working principle of the rechargeable ZABs, it is described in detail as following: the discharging process involves the coupling of the cathodic oxygen reduction reaction (ORR) and Zn anodization reaction. Typically, $O_2$ diffuses into the air cathode, and then is reduced to $OH^-$ ions, the generated $OH^-$ ions migrate to the Zn anode. Meanwhile, metal Zn is oxidized to release $Zn^{2+}$ ions, which then combine with the migrated $OH^-$ ions to form $[Zn(OH)_4]^{2-}$. When the concentration of $Zn(OH)_4]^{2-}$ in the electrolyte reaches a saturated state, it will decompose into ZnO products. During the charging process, it involves the oxygen evolution reaction (OER, the reverse reaction of the ORR) and the corresponding charge process is as follows: $OH^-$ ions are oxidized to $O_2$ at the cathode and the $Zn^{2+}$ ions in the electrolyte are reduced to metallic Zn and deposited on the anode surface. The complete discharge/charge processes of ZABs can be illustrated as the below equations:

Anode: $$Zn + 4OH^- \leftrightarrow [Zn(OH)_4]^{2-} + 2e^- \quad (7)$$

Cathode: $$O_2 + 2H_2O + 4e^- \leftrightarrow 4OH^- \quad (8)$$

Overall reaction: $$2Zn + O_2 \leftrightarrow 2ZnO \quad (9)$$

**DFT Computational methods:** All the density functional theory (DFT) calculations were performed by using the plane-wave method with Vienna Ab Initio Simulation Package (VASP).[1,2]The frozen-core projector augmented wave (PAW)[3] potential and generalized gradient approximation in the Perdew-Burke-Ernzerhof (PBE) functional were adopted to simulate the electron exchange and correlation energy.[4] The cutoff energy for plane wave basis expansion was fixed as 400 eV to describe the interaction between core electrons and valence electrons. The force

on all atoms in each structure was controlled less than 0.01 eV/Å for convergence criterion of geometry relaxation. The Zero damping DFT-D3 method[5] of Grimme was used to describe the long-range Van der Waals interactions between atoms. The self-consistent calculations apply a convergence energy threshold of $10^{-5}$ eV. And a Γ-centered Monkhorst-Pack k-point scheme of 3 × 3 × 1 mesh grid was utilized for all structural optimizations. All structure models were constructed by a 6 × 6 × 1 hexagonal graphene, the dimension of the supercell is 14.76 × 12.78 × 15.00 $Å^3$ along x-, y-, and z- directions, respectively. And the vacuum space was chosen as 15 Å to avoid artificial interactions between periodic structures in z direction.

The free energy of each elementary step in the proton coupled electron transfer reactions was computed using the computational hydrogen electrode (CHE) model for both ORR and OER. For ORR calculation in this work, the general four electrons pathway occur in alkaline media as the following steps:

$$M^* + O_2(g) + H_2O(l) + e^- \rightarrow OOH^* + OH^- \quad (10)$$

$$OOH^* + e^- \rightarrow O^* + OH^- \quad (11)$$

$$O^* + H_2O(l) + e^- \rightarrow OH^* + OH^- \quad (12)$$

$$OH^* + e^- \rightarrow M^* + OH^- \quad (13)$$

For OER, the pathway goes through the steps as below:

$$M^* + OH^- \rightarrow OH^* + e^- \quad (14)$$

$$OH^* + OH^- \rightarrow O^* + H_2O(l) + e^- \quad (15)$$

$$O^* + OH^- \rightarrow OOH^* + e^- \quad (16)$$

$$OOH^* + OH^- \rightarrow M^* + O_2(g) + H_2O(l) + e^- \quad (17)$$

Where * represents the active site on the exposed catalyst surface and OH*, O*, and OOH* denote adsorbed intermediates.

For each single step, the Gibbs free energy ΔGi (i = 1, 2, 3, 4) can be calculated according to the equation:

$$\Delta Gi = \Delta E + \Delta ZPE - T\Delta S \quad (18)$$

Where ΔE is the total energy difference between the reactants and products in these reactions, ΔZPE means the zero-point energy (ZPE) correction.

The thermodynamic overpotential η ORR/OER for a certain material can be determined by:

$$\Delta GORR/OER = \max\{\Delta G1, \Delta G2, \Delta G3, \Delta G4\} \quad (19)$$

$$\eta ORR/OER = \Delta GORR/OER/e - 1.23\ V \quad (20)$$

where ΔG1, ΔG2, ΔG3 and ΔG4 are the Gibbs free energy for each elementary step of ORR/OER pathway.

## II. Supplementary figures and tables

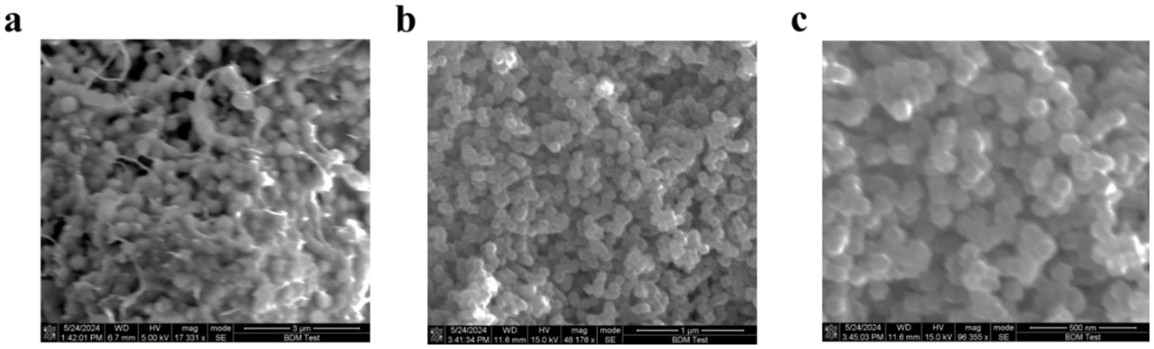


**Figure S1.** The SEM images of (a) FeNi-NC@MWCNTs, (b) and (c) for FeNi-NC.

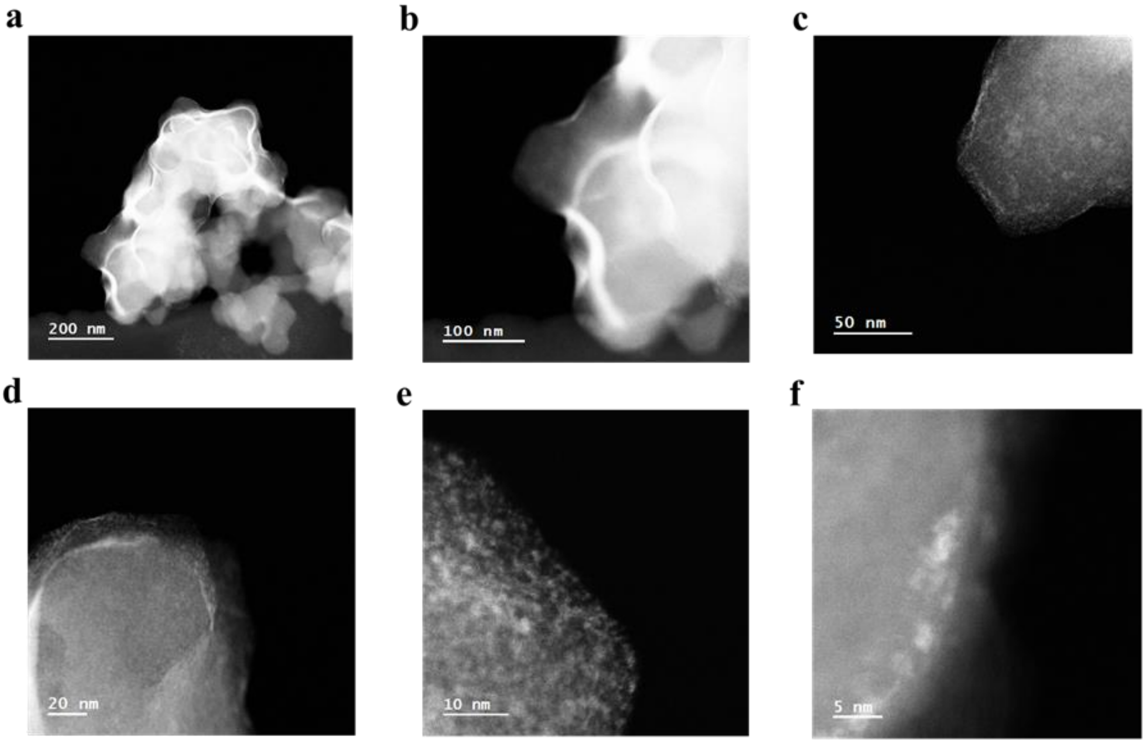


**Figure S2.** The aberration-corrected HAADF-STEM images of FeNi-NC@MWCNTs.

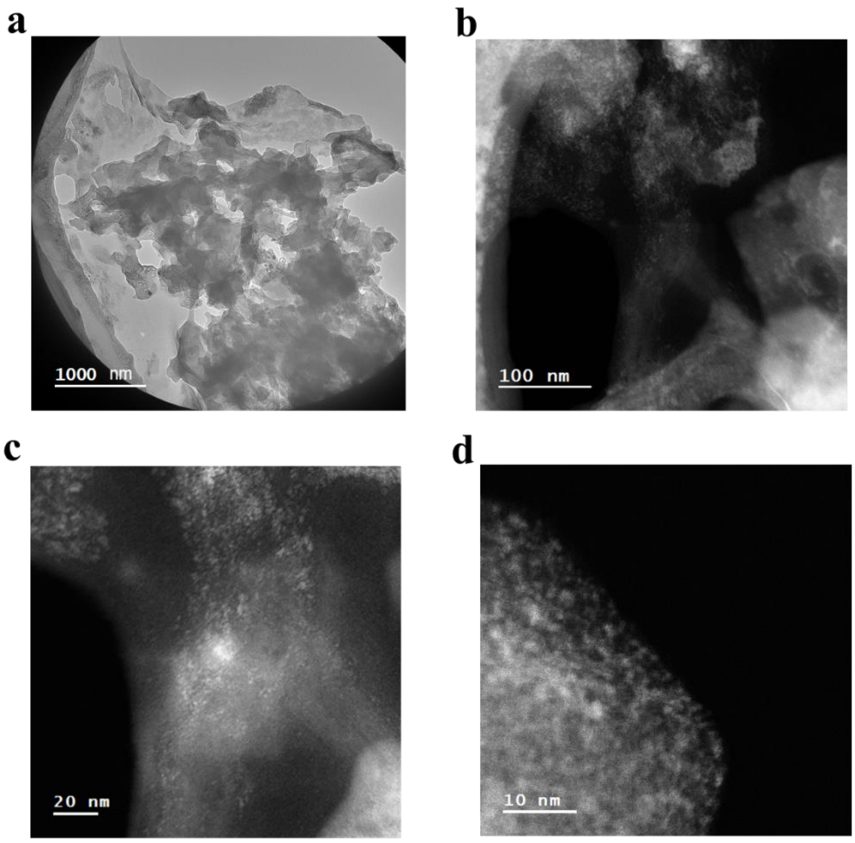


**Figure S3.** The aberration-corrected HAADF-STEM images of FeNi-NC.

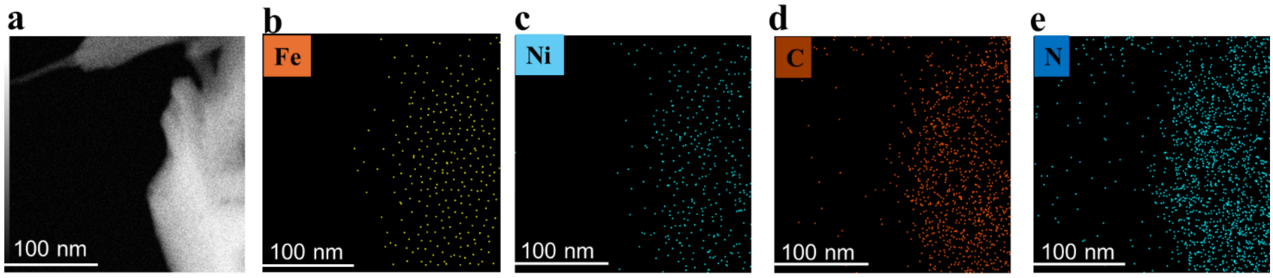


**Figure S4.** EDS mapping patterns for C, N, Fe and Ni of the FeNi-NC@MWCNT catalyst.

**Table S1.** Metal element contents of FeNi-NC@MWCNTs and FeNi-NC determined by ICP-OES

| Catalyst materials | ICP-OES | |
|---|---|---|
| | Fe (wt. %) | Ni (wt. %) |
| **FeNi-NC@MWCNT** | 0.57 | 0.62 |
| **FeNi-NC** | 0.45 | 0.38 |

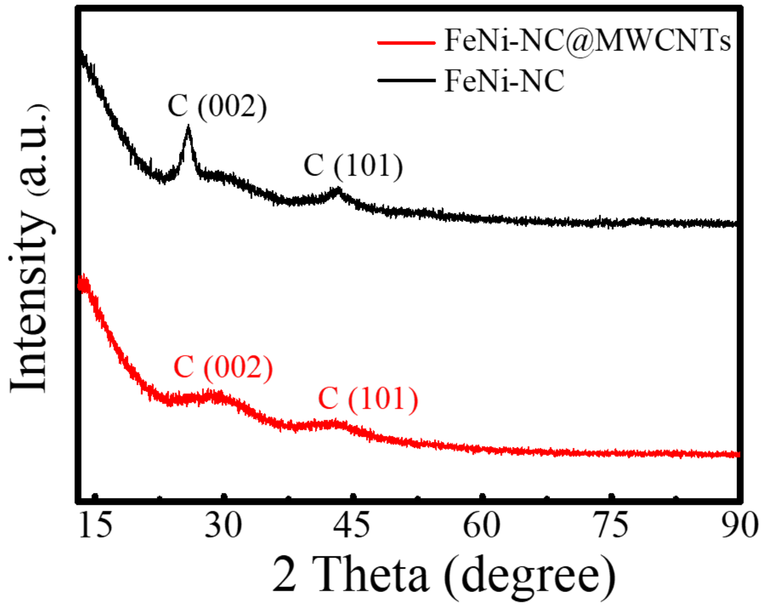


**Figure S5**. The XRD pattern of FeNi-NC@MWCNTs and FeNi-NC.

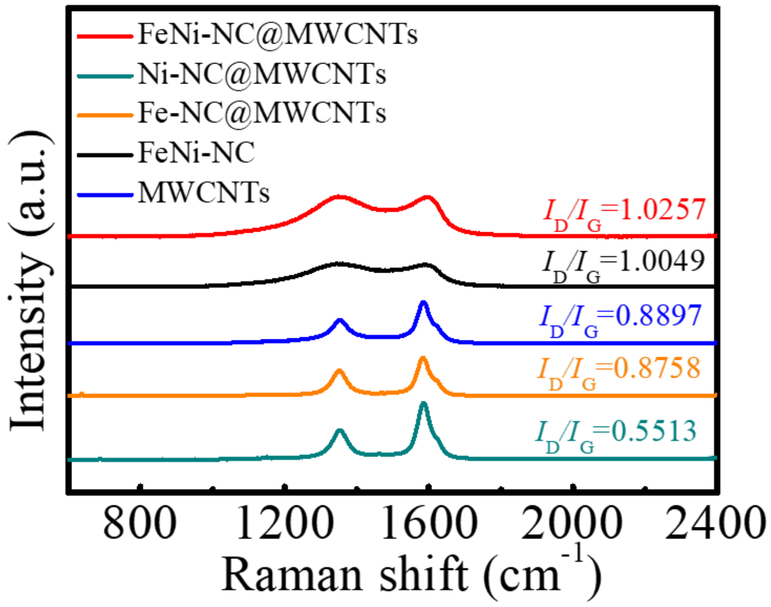


**Figure S6**. The Raman spectra of FeNi-NC@MWCNTs, Fe-NC@MWCNTs, Ni-NC@MWCNTs, FeNi-NC and MWCNTs.

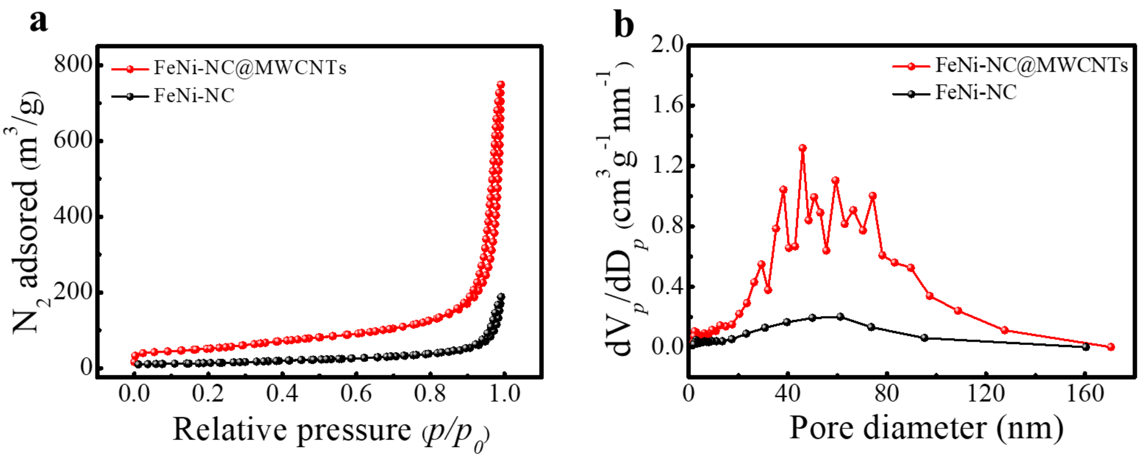


**Figure S7**. (a) Nitrogen adsorption-desorption isotherms of FeNi-NC@MWCNTs and FeNi-NC. (b) Pore-size distributions.

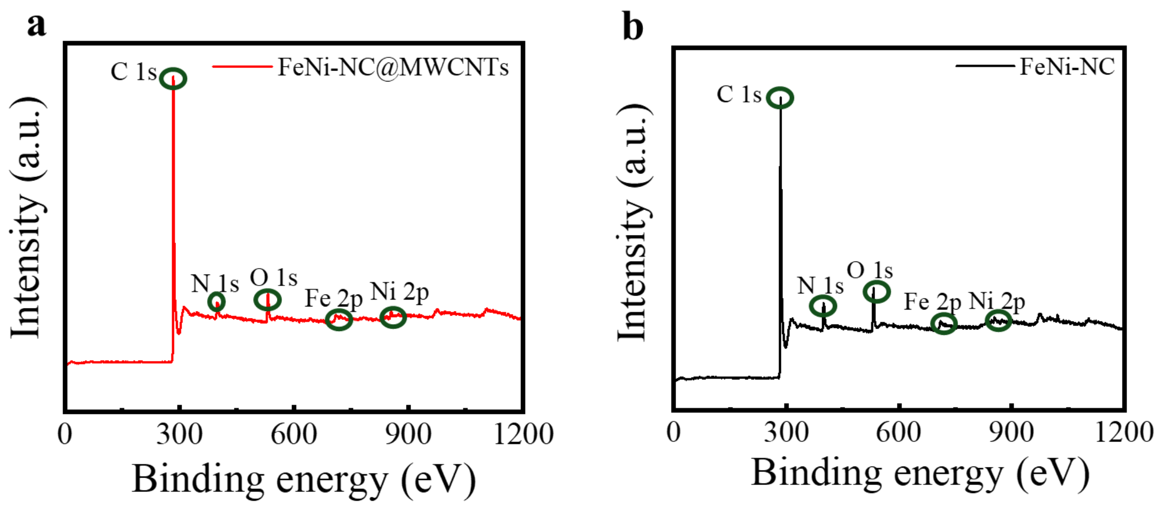


**Figure S8**. Survey XPS of FeNi-NC@MWCNTs (a) and FeNi-NC (b), revealing the presence of C, N, Fe and Ni.

**Table S2**. The Fe K-edge EXAFS curve fitting parameters and the Ni K-edge EXAFS curve fitting parameters.

| FeNi-NC@MWCNTs | Shell | C.N. | R(Å) | $\sigma^2$ ($Å^2$) | R factor |
|---|---|---|---|---|---|
| Fe K-edge | Fe-N | 2.62 ± 0.524 | 2.12 ± 0.021 | 0.0005 ± 0.0001 | $1.0 \times 10^{-2}$ |
| | Fe-Ni | 1.31 ± 0.262 | 2.51 ± 0.025 | 0.0030 ± 0.0006 | |
| Ni K-edge | Ni-N | 3.58 ± 0.71 | 2.09 ± 0.021 | 0.01243 ± 0.00248 | $1.9 \times 10^{-3}$ |
| | Ni-Fe | 1.79 ± 0.35 | 2.51 ± 0.025 | 0.00515 ± 0.00103 | |

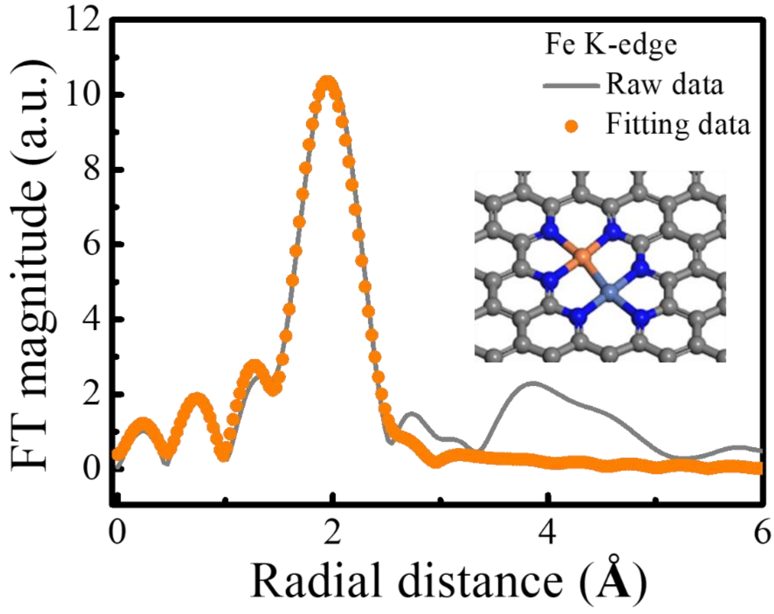


**Figure S9**. (a) Experimental and fitted FT-EXAFS curves at Fe K-edge of FeNi-NC@MWCNTs in R space and the fitting curves based on the monomeric FeNi-$N_6$ model (inset).

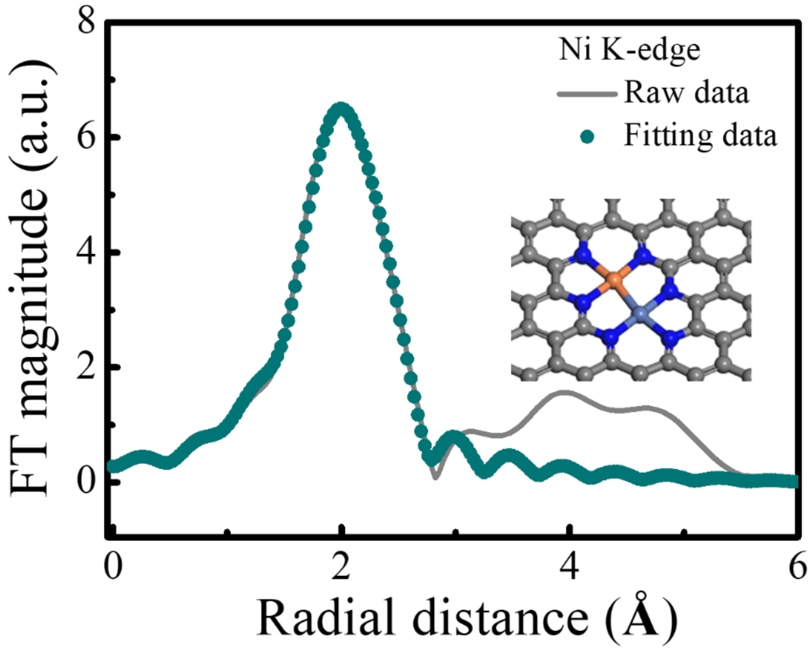


**Figure S10**. (a) Experimental and fitted FT-EXAFS curves at Ni K-edge of FeNi-NC@MWCNTs in R space and the fitting curves based on the monomeric FeNi-$N_6$ model (inset).

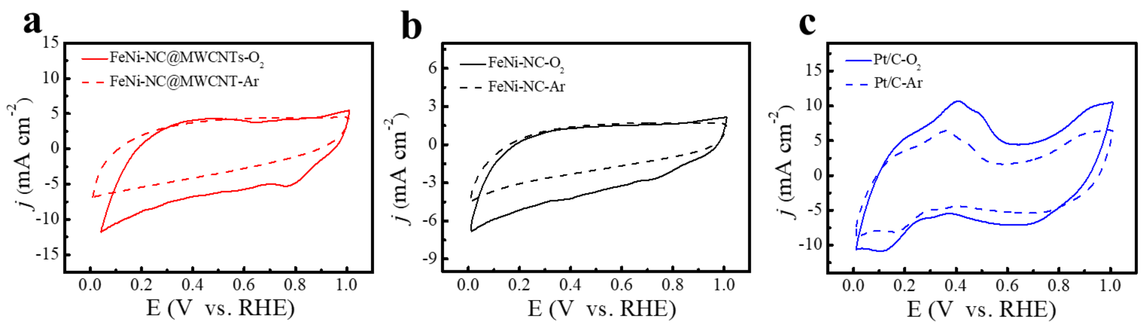


**Figure S11.** CV curves of FeNi-NC@MWCNTs, FeNi-NC, and Pt/C at scan rate of 0.1 V $s^{-1}$. The solid and dashed lines correspond to the measurement in $O_2$- and Ar-saturated 0.1M KOH solution, respectively.

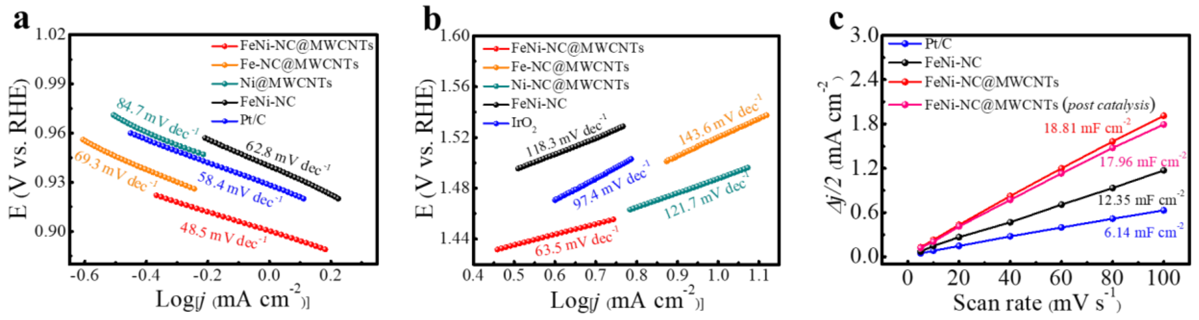


**Figure S12.** (a) ORR Tafel plots of FeNi-NC@MWCNT, Fe-NC@MWCNTs, Ni-NC@MWCNTs, FeNi-NC and commercial Pt/C. (b) OER Tafel plots of FeNi-NC@MWCNT, Fe-NC@MWCNTs, Ni-NC@MWCNTs, FeNi-NC and commercial Pt/C. (c) Half of the capacitive current density ($\Delta j/2$) at 1.1 V (vs. RHE) as a function of the scan rates of FeNi-NC@MWCNT, FeNi-NC, Pt/C and FeNi-NC@MWCNT post OER catalysis.

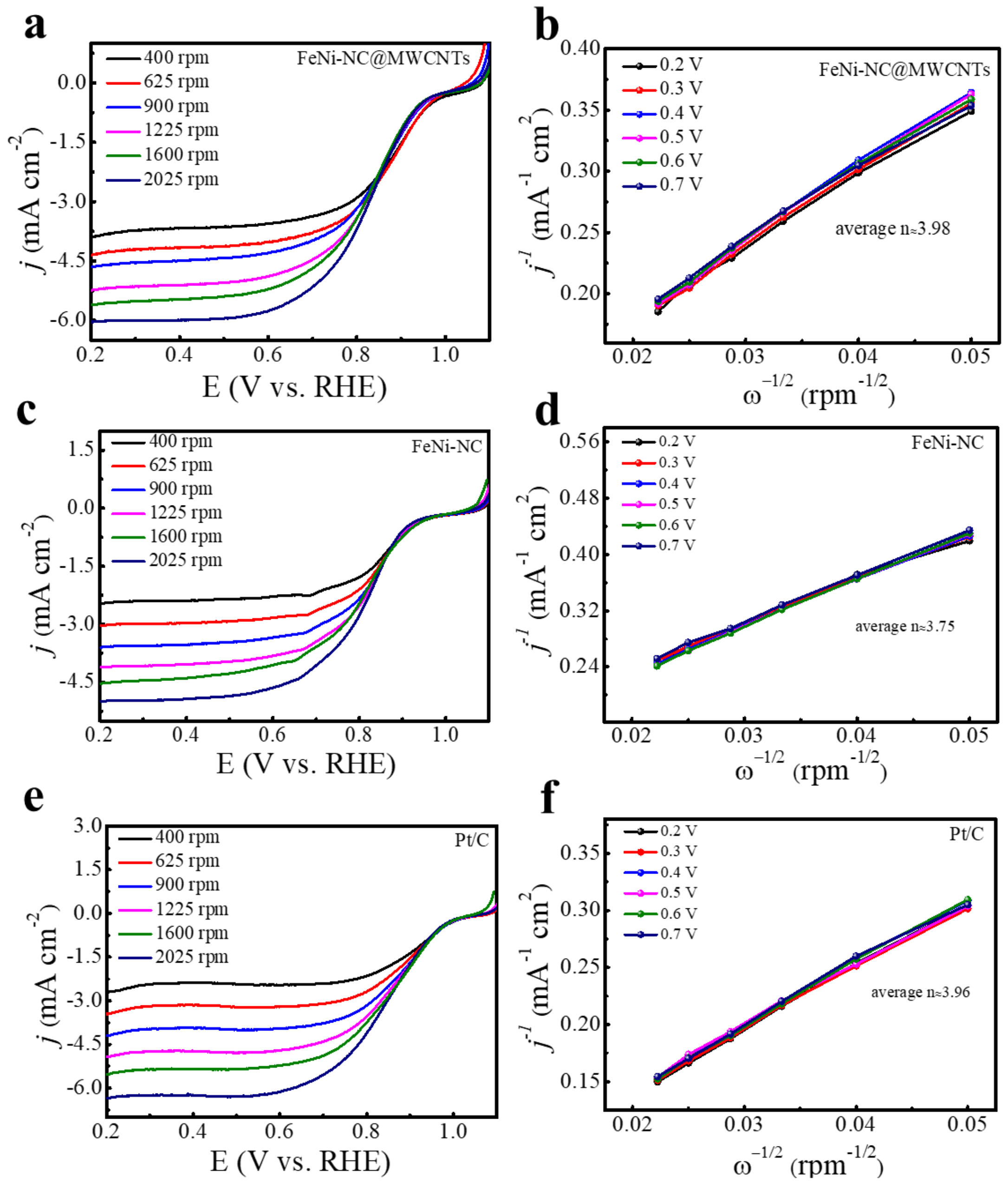


**Figure S13.** ORR LSV curves of FeNi-NC@MWCNTs, FeNi-NC, and Pt/C at different rotating rates (a, c, e) corresponding K–L plot and electron transfer numbers (b, d and f).

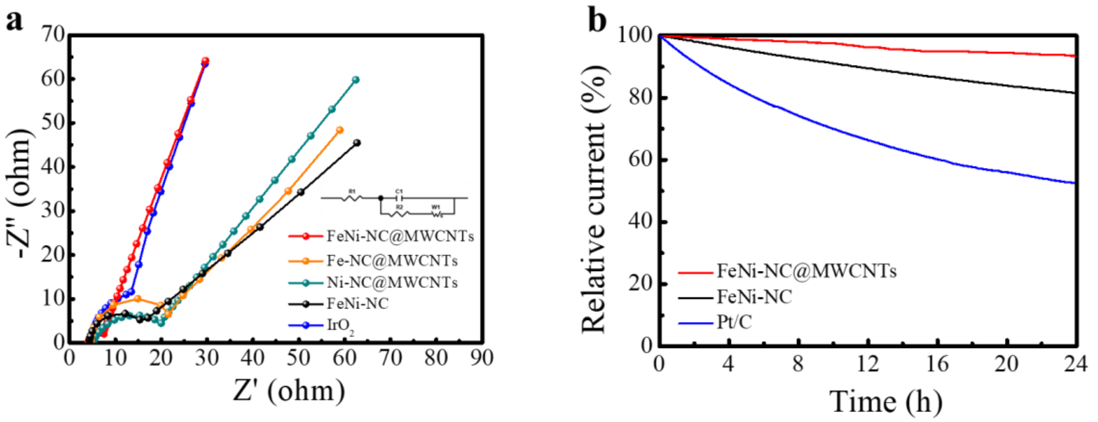


**Figure S14**. (a) The Nyquist plot for FeNi-NC@MWCNTs, Fe-NC@MWCNTs, Ni-NC@MWCNTs, FeNi-NC and $IrO_2$ in 0.1 M KOH solution. In this study, the electrochemical impedance spectroscopy (EIS) measurements were conducted at the potential of 1.56V. (insert: equivalent circuit). (b) The Chronoamperometry curves of FeNi-NC@MWCNTs, FeNi-NC and Pt/C at 0.8 V vs. RHE in $O_2$ saturated electrolyte for ORR stability test.

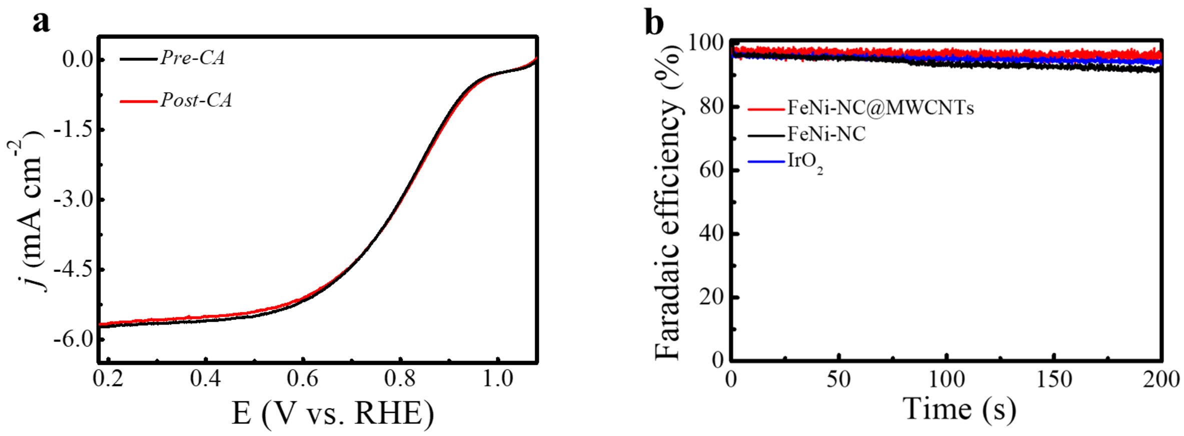


**Figure S15**. (a) The ORR polarization curves of FeNi-NC@MWCNTs before and after chronoamperometry test. (b) The OER faradaic efficiency of FeNi-NC@MWCNTs, FeNi-NC and $IrO_2$ for OER were determined by RRDE analysis.

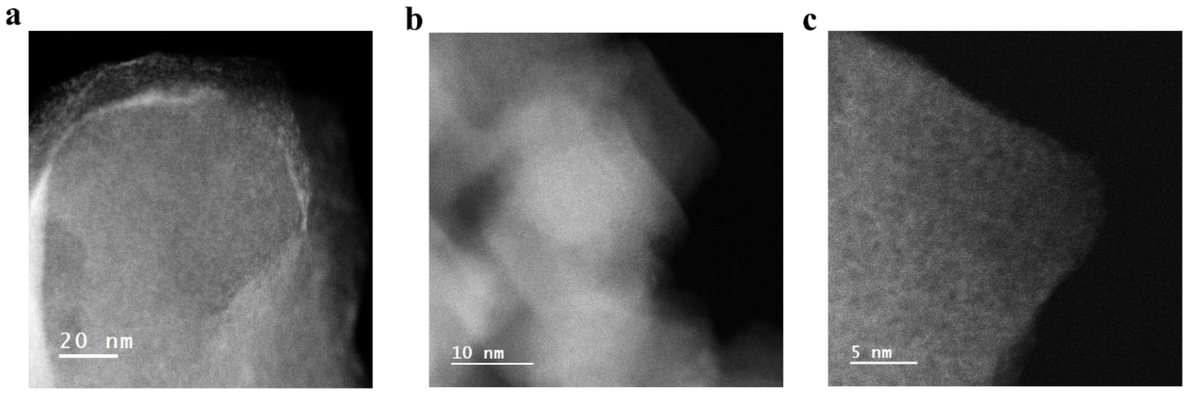


**Figure S16**. The aberration-corrected HAADF-STEM images of FeNi-NC@MWCNTs after ORR chronoamperometric test.

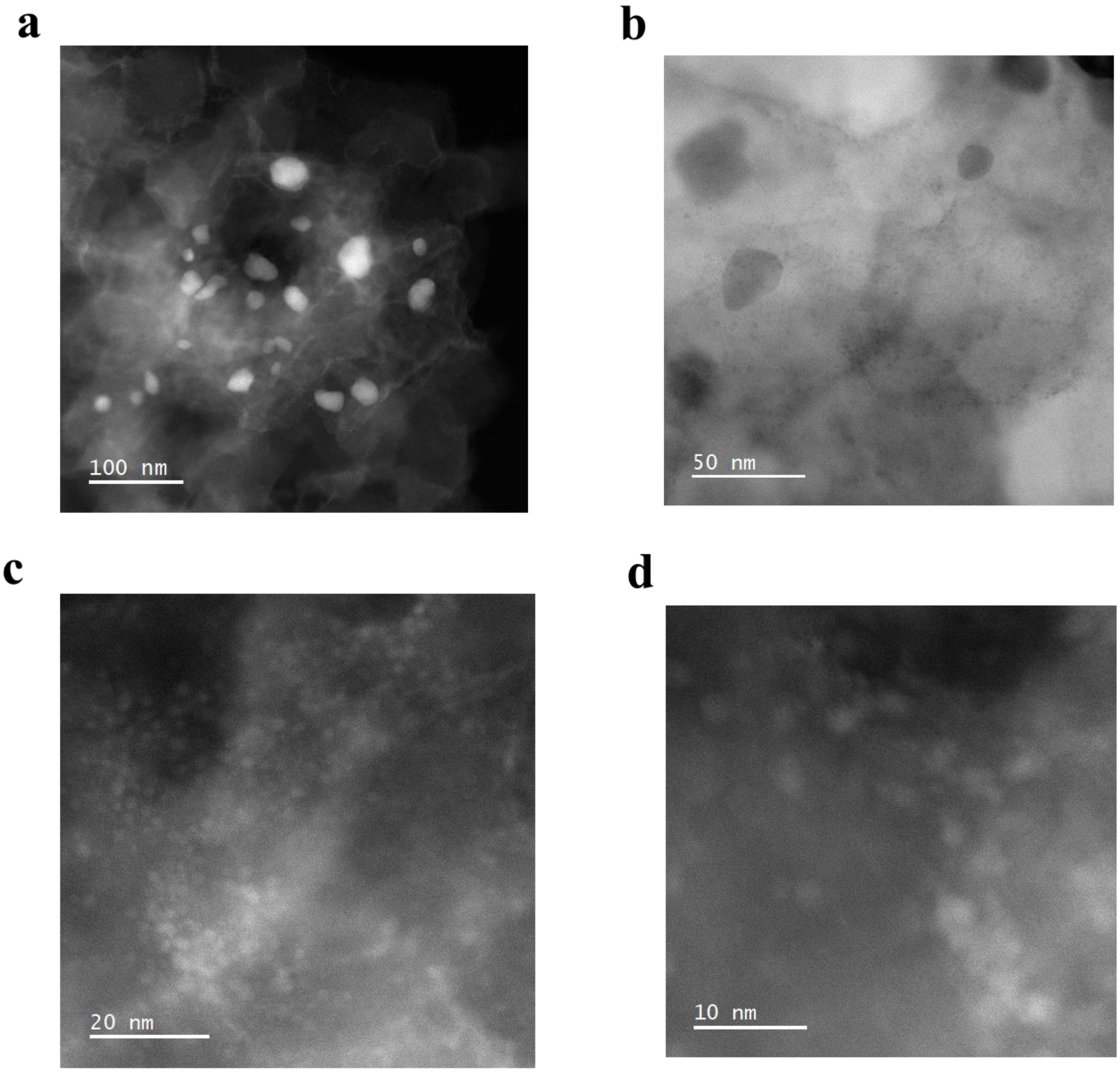


**Figure S17**. The aberration-corrected HAADF-STEM images of FeNi-NC@MWCNTs after OER chronoamperometric test.

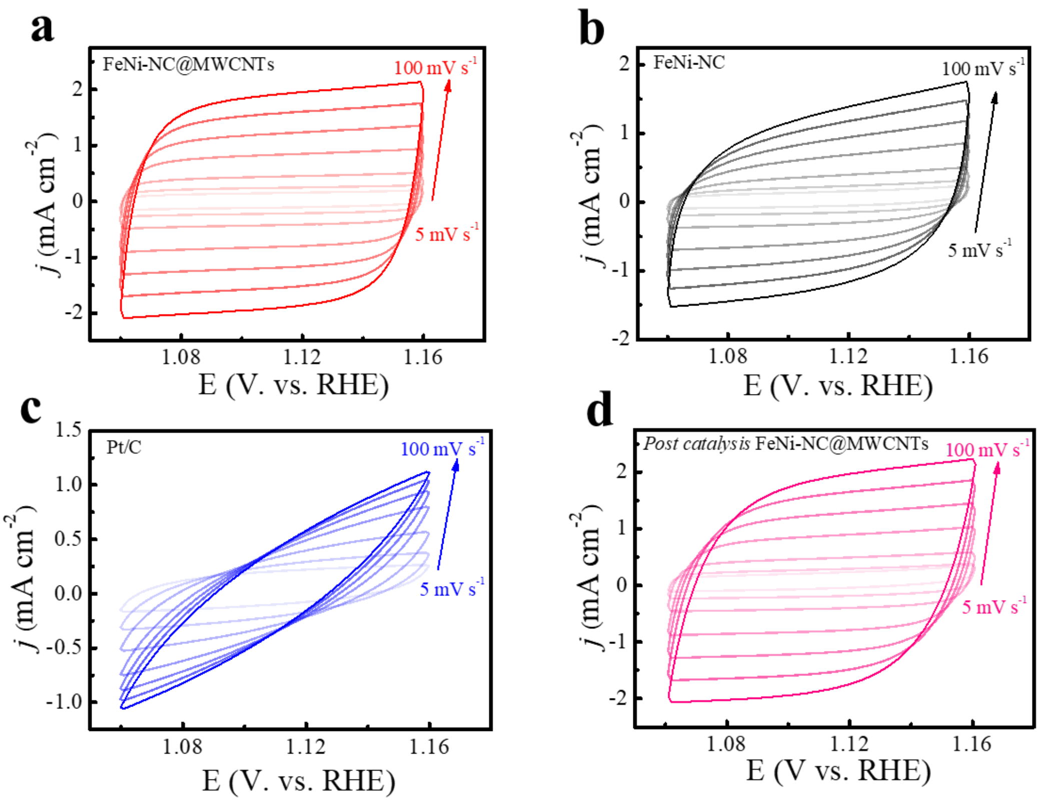


**Figure S18**. Double-layer capacitance measurements for determining electrochemically active surface area of the catalysts. CV curves of (a) FeNi-NC@MWCNTs, (b) FeNi-NC (c) Pt/C, and (d) FeNi-NC@MWCNTs post OER catalysis respectively. The CVs were scanned from 1.06 to 1.16 V vs. RHE with the scan rates from 5 to 100 mV $s^{-1}$.

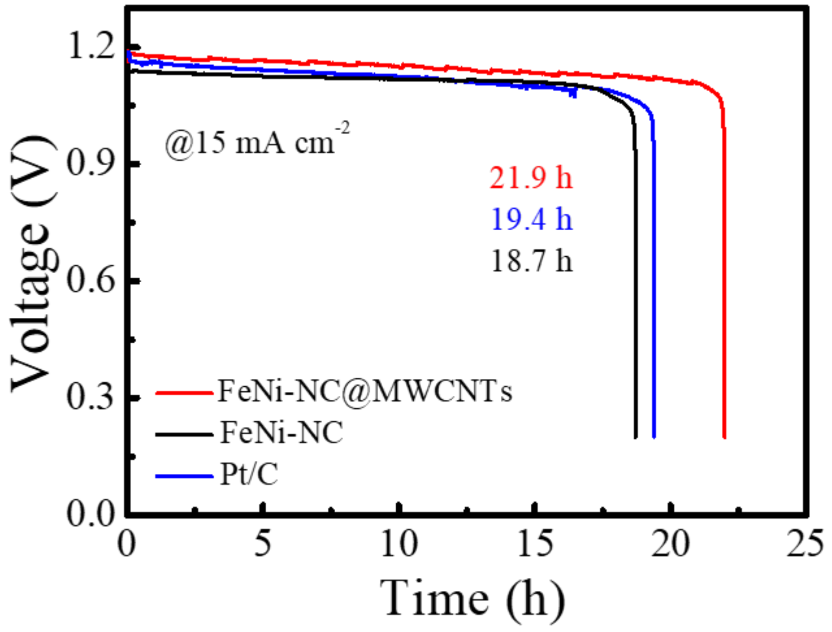


**Figure S19**. Galvanostatic discharge curves of ZABs with different catalysts at a constant current density of 15 mA $cm^{-2}$.

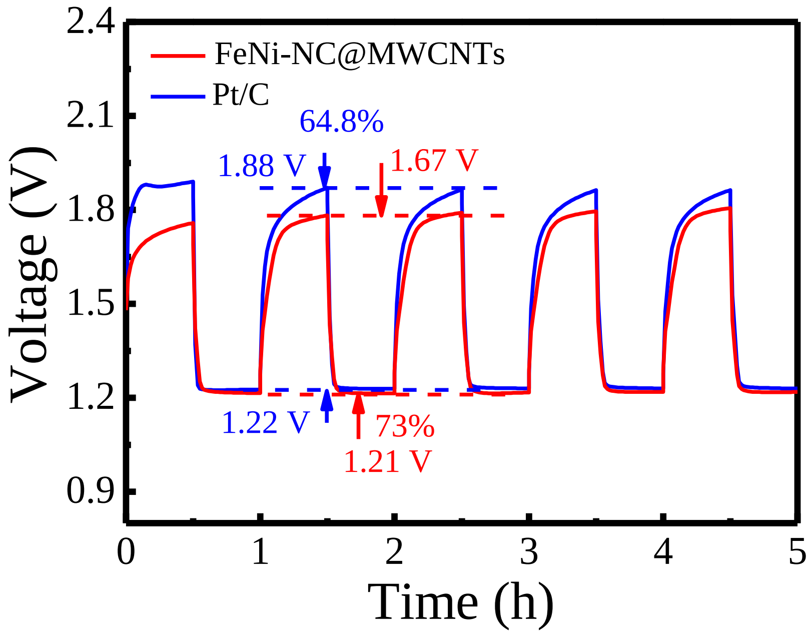


**Figure S20**. The charge-discharge profiles of ZABs driven by FeNi-NC@MWCNTs and Pt/C catalysts with a duration of 1 h per cycle. The round-trip efficiency was calculated according to the cut-off voltage of the second cycle.

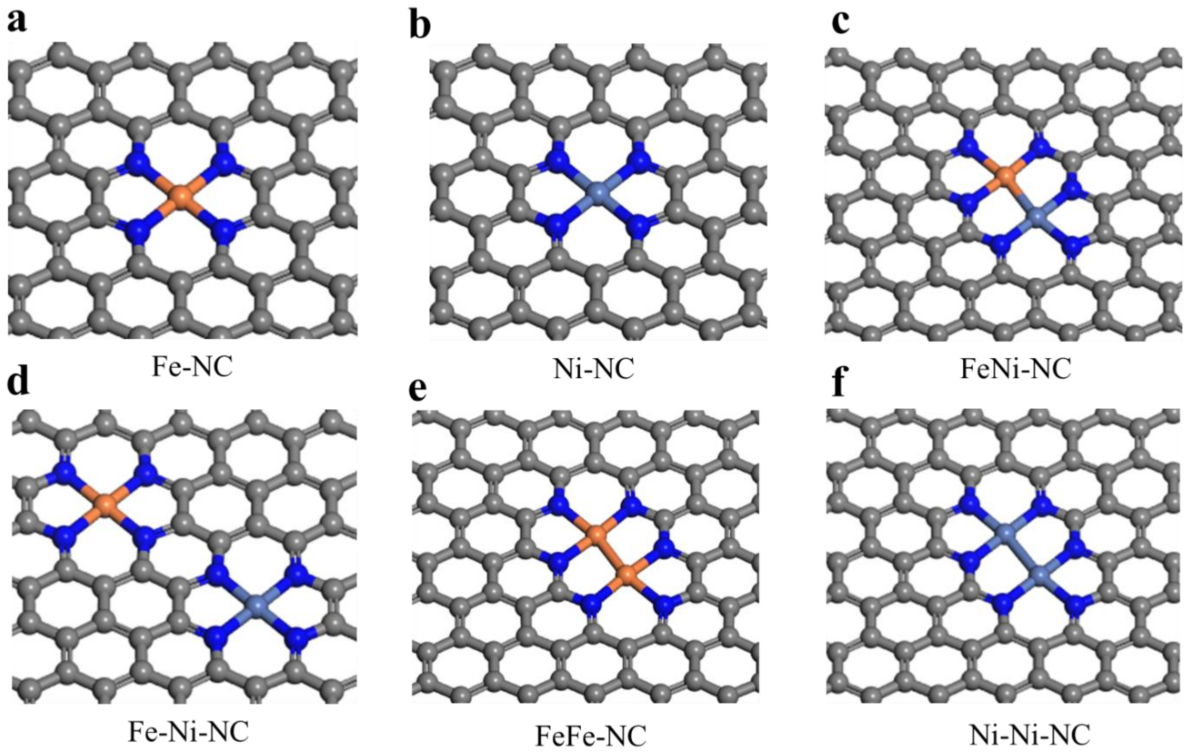


**Figure S21**. Initial unit cell of Fe-NC (a), Ni-NC (b), FeNi-NC (c), Fe-Ni-NC (d), FeFe-NC (e) and NiNi-NC (f). The grey, blue, orange, and cyan balls represent carbon, nitrogen, iron, and nickel atoms, respectively.

**Table S3.** Adsorption energy of OH* ($\Delta G_{OH*}$) on active sites in different models Fe-NC, Ni-NC, FeNi-NC, Fe-Ni-NC, FeFe-NC and NiNi-NC, respectively.

| | Fe-NC | Ni-NC | FeNi-NC | Fe-Ni-NC |
|---|---|---|---|---|
| $\Delta G_{OH*}$ | 0.20 | 1.67 | -0.24 | 0.19 |

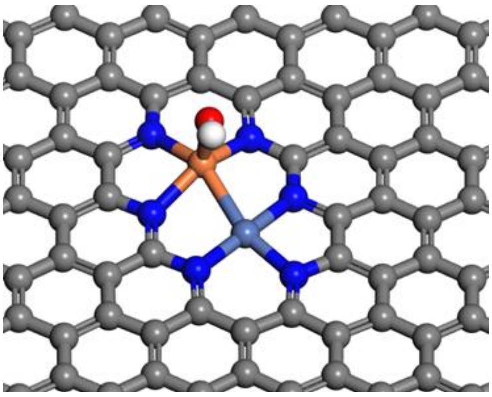

**Figure S22**. Unit cell after adsorption of *OH on FeNi-NC model (FeNi-OH-NC). The grey, blue, orange, and cyan balls represent carbon, nitrogen, iron, and nickel atoms, respectively.

**Table S4.** Formation energy of oxygen intermediates adsorbed on active sites in different models Fe-NC, Ni-NC, FeNi-NC, Fe-Ni-NC, FeFe-NC, NiNi-NC and FeNi-OH-NC, respectively.

| | Fe-NC | Ni-NC | Fe@Fe-Ni-NC | Ni@Fe-Ni-NC | FeFe-NC | NiNi-NC | FeNi-NC | FeFe-OH-NC | FeNi-OH-NC |
|---|---|---|---|---|---|---|---|---|---|
| $\Delta G_{OH*}$ | 0.20 | 1.67 | 0.19 | 1.65 | -0.62 | 1.97 | -0.24 | 0.50 | 0.82 |
| $\Delta G_{O*}$ | 1.04 | 2.47 | 1.07 | 2.46 | 0.52 | 1.11 | 0.64 | 0.99 | 1.82 |
| $\Delta G_{OOH*}$ | 1.86 | 0.34 | 1.83 | 0.35 | 2.85 | 2.23 | 2.51 | 2.10 | 1.38 |
| $\Delta G_{O2*}$ | 1.82 | 0.44 | 1.83 | 0.46 | 2.17 | -0.39 | 2.01 | 1.33 | 0.90 |

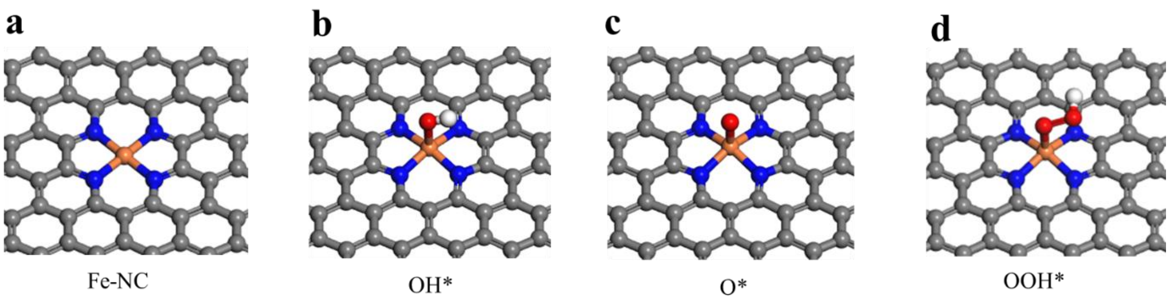


**Figure S23**. Unit cell before (a) and after adsorption of *OH (b), *O (c) and OOH* (d) on Fe-NC model. The grey, blue, and orange balls represent carbon, nitrogen and iron atoms, respectively.

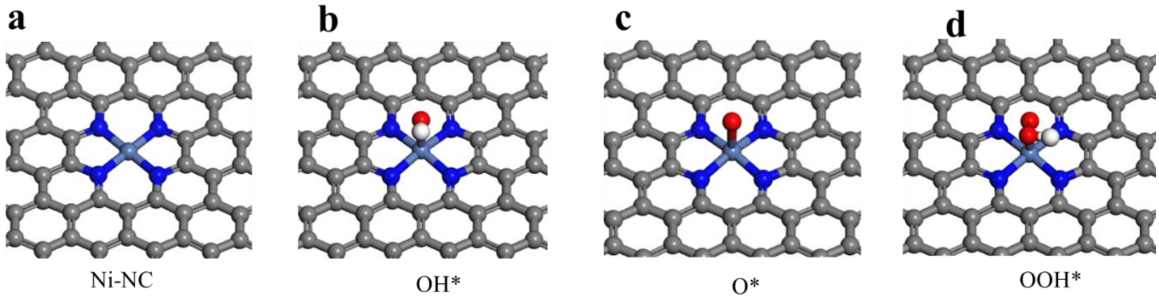


**Figure S24**. Unit cell before (a) and after adsorption of *OH (b), *O (c) and OOH* (d) on Ni-NC model. The grey, blue, and cyan balls represent carbon, nitrogen, and nickel atoms, respectively.

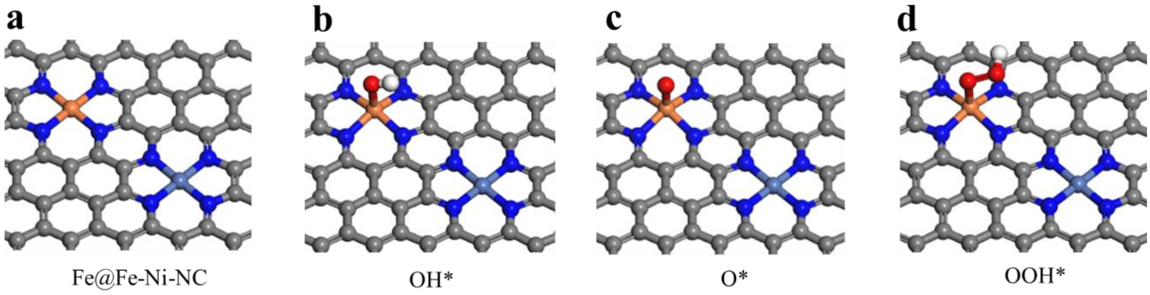


**Figure S25**. Unit cell before (a) and after adsorption of *OH (b), *O (c) and OOH* (d) on Fe@Fe-Ni-NC model. The grey, blue, orange, and cyan balls represent carbon, nitrogen, iron, and nickel atoms, respectively.

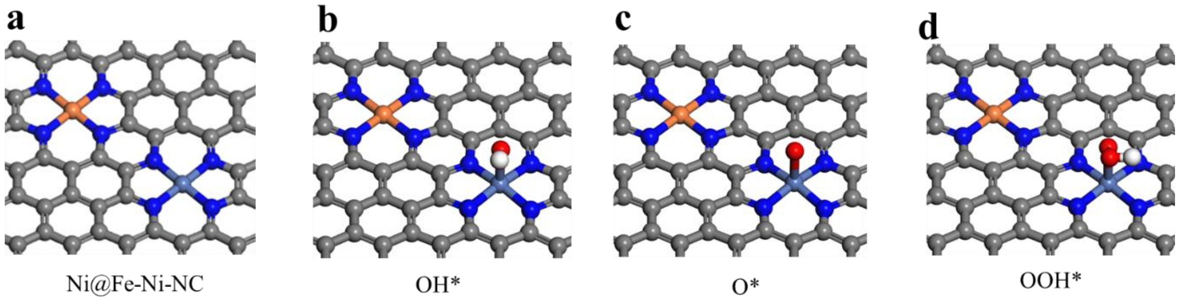


**Figure S26**. Unit cell before (a) and after adsorption of *OH (b), *O (c) and OOH* (d) on Fe@Fe-Ni-NC model. The grey, blue, orange, and cyan balls represent carbon, nitrogen, iron, and nickel atoms, respectively.

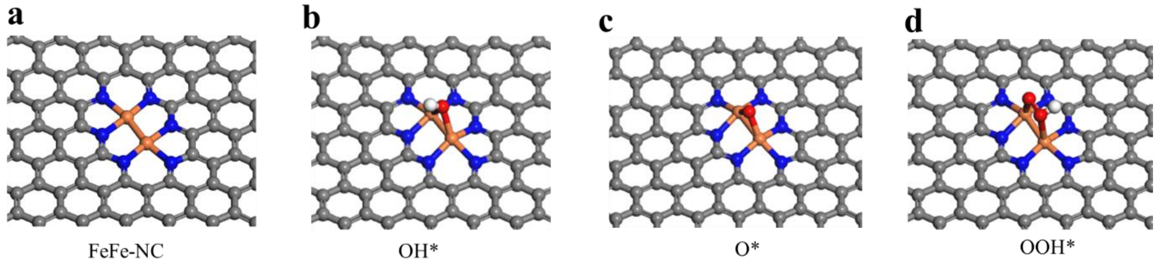


**Figure S27**. Unit cell before (a) and after adsorption of *OH (b), *O (c) and OOH* (d) on FeFe-NC model. The grey, blue, and orange balls represent carbon, nitrogen, and iron atoms, respectively.

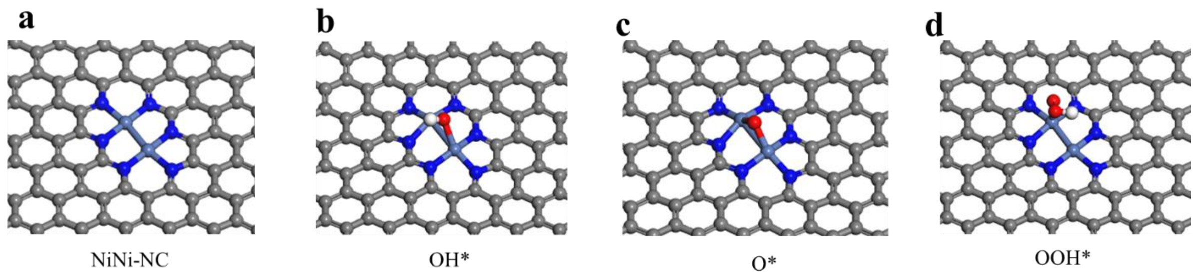


**Figure S28**. Unit cell before (a) and after adsorption of *OH (b), *O (c) and OOH* (d) on NiNi-NC model. The grey, blue, and cyan balls represent carbon, nitrogen, and nickel atoms, respectively.

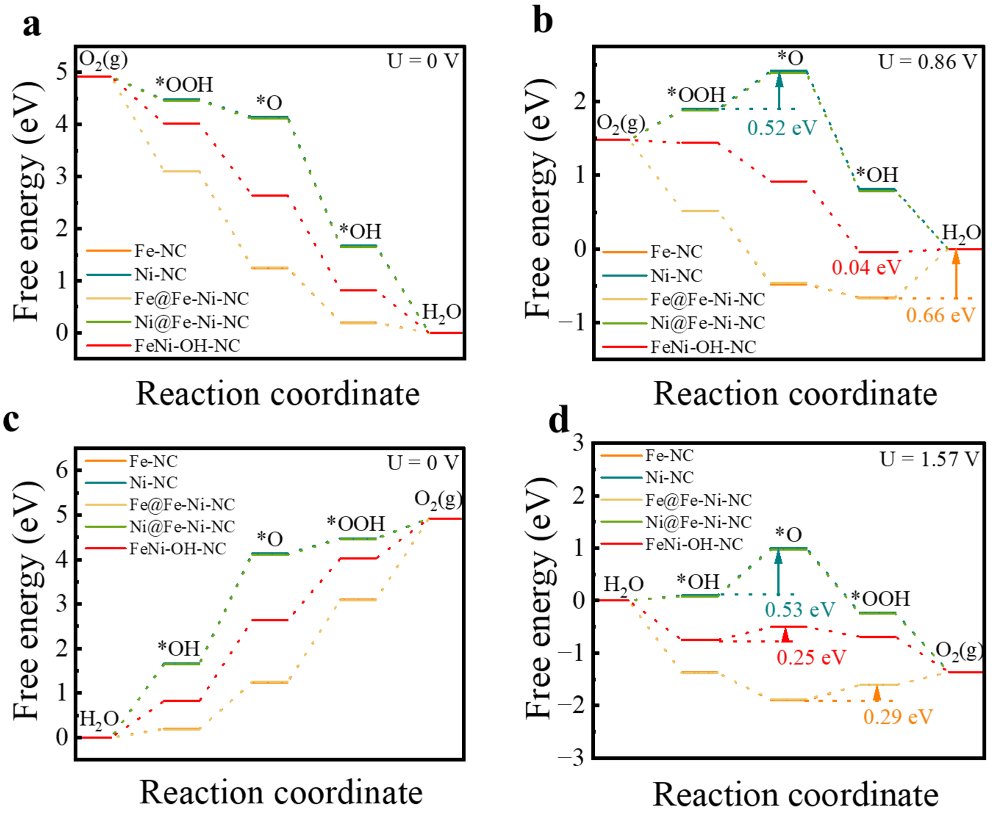

**Figure S29**. Free energy diagrams of ORR intermediates on Fe-NC, Ni-NC, FeNi-OH-NC and Fe-Ni-NC models at potential of 0 V (a) and 0.86 V (b). Free energy diagrams of OER intermediates on Fe-NC, Ni-NC, FeNi-OH-NC and Fe-Ni-NC modes at potential 0 V (c) and 1.57 V (d).

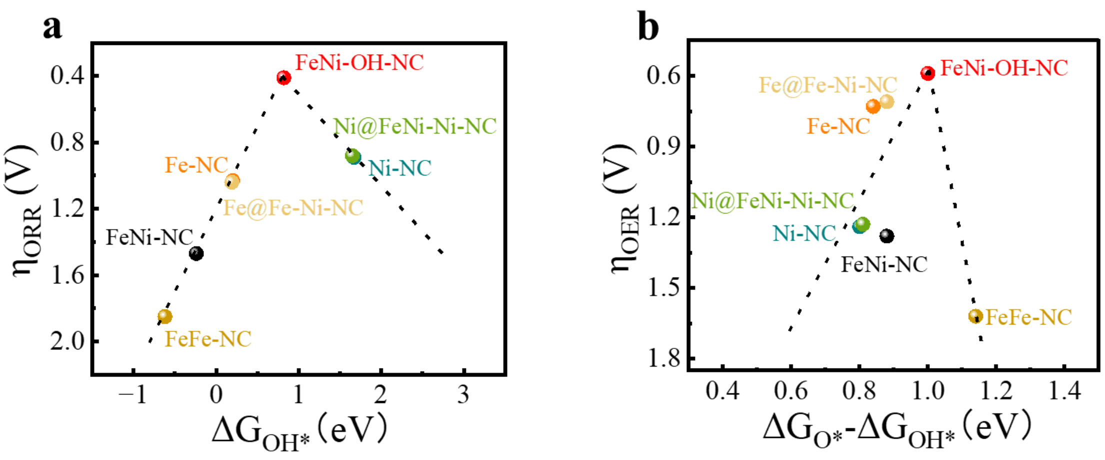


**Figure S30**. (a) Volcano plot of overpotential η of ORR versus $\Delta G_{*OH}$ for Fe-NC, Ni-NC, FeNi-OH-NC and Fe-Ni-NC models. (b) Volcano plot of overpotential η of OER versus $\Delta G_{O*}$ -$\Delta G_{OH*}$ for Fe-NC, Ni-NC, FeNi-OH-NC and Fe-Ni-NC models.

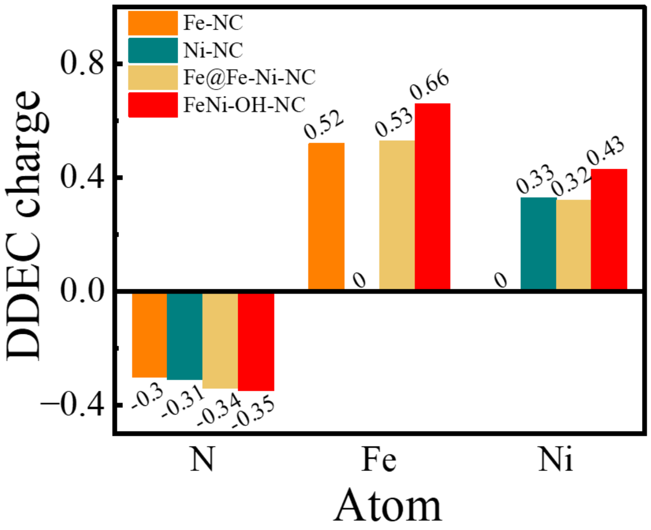


**Figure S31.** Density derived electrostatic and chemical (DDEC) charge distributions of the constructed models Fe-NC, Ni-NC, Fe@Fe-Ni-NC and FeNi-OH-NC on Fe, Ni and N, respectively. The Bader effective charge of N is given as the average value.

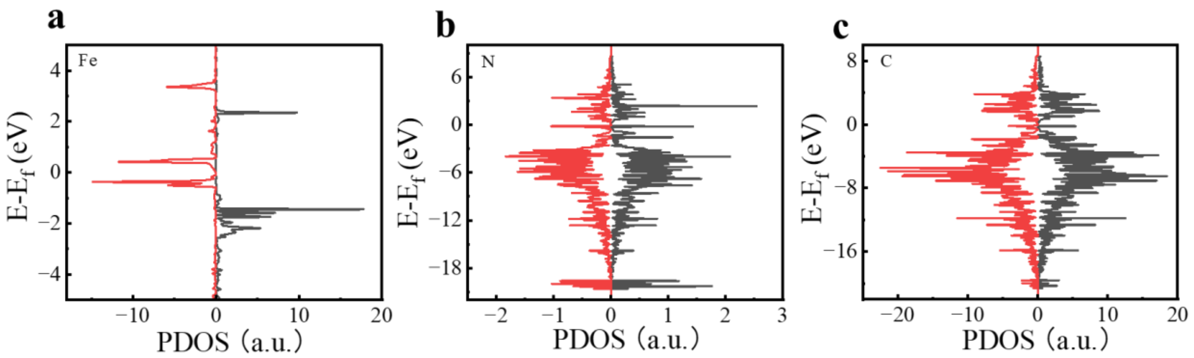


**Figure S32**. Projected density of states of metal Fe d orbitals and non-metal (N and C) p orbitals for the Fe-NC model.

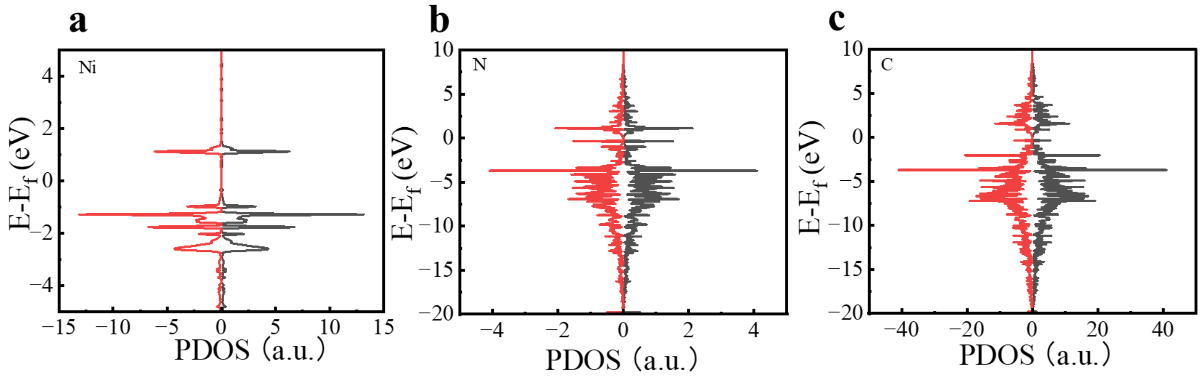


**Figure S33**. Projected density of states of metal Ni d orbitals and non-metal (N and C) p orbitals in the Ni-NC model.

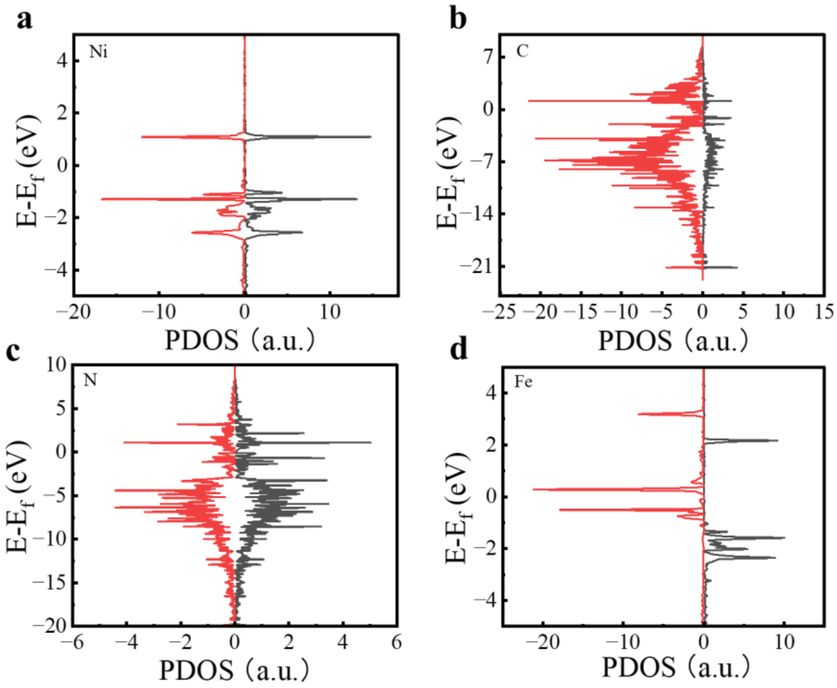


**Figure S34**. Projected density of states of metal (Fe and Ni) d orbitals and non-metal (N and C) p orbitals in the Fe-Ni-NC model.

**Table S5.** Comparison of the ORR and OER performance between FeNi-NC@MWCNTs and other recently reported single/dual atom catalysts.

| Catalysts | $E_{1/2}$ (V vs. RHE ) | $E_{j=10}$ (V vs. RHE) | $\Delta E$ ($E_{j=10}$-$E_{1/2}$) (V vs. RHE) | Electrolyte | Reference |
|---|---|---|---|---|---|
| FeNi-NC@MWCNTs | 0.9 | 1.61 | 0.71 | 0.1 KOH | This work |
| $FeN_4B$-$NiN_4B$ | 0.9 | 1.618 | 0.718 | 0.1 KOH | [6] |
| P doped Fe-NC | 0.85 | 1.64 | 0.79 | 0.1 KOH | [7] |
| Pd/$WO_{2.72}$ | 0.86 | 1.59 | 0.73 | 0.1 KOH | [8] |
| NiFe LDH-A Fe/NC-CNT | 0.84 | 1.59 | 0.78 | 0.1 KOH | [9] |
| SAC(Fe, Ni, Zn) | 0.88 | 1.63 | 0.75 | 0.1 KOH | [10] |
| FeNi-HPNC | 0.868 | 1.59 | 0.72 | 0.1 KOH | [11] |
| PA@Z8-Fe-N-C | 0.88 | 1.77 | 0.89 | 0.1 KOH | [12] |
| FeCo-DACs/NC | 0.877 | 1.6 | 0.723 | 0.1 KOH | [13] |
| CoNC SAC | 0.86 | 1.65 | 0.79 | 0.1 KOH | [14] |
| Fe,Co,N-C | 0.9 | 1.64 | 0.74 | 0.1 KOH | [15] |
| $Fe_1Co_1$-CNF | 0.86 | 1.73 | 0.87 | 0.1 KOH | [16] |
| MS-CoSA-N-C | 0.86 | 1.60 | 0.76 | 0.1 KOH | [17] |
| Ni-N4/GHSs/ Fe-N4 | 0.83 | 1.62 | 0.79 | 0.1 KOH | [18] |
| FeCo-NPC-1100 | 0.79 | 1.60 | 0.81 | 0.1 KOH | [19] |
| FeNi/N−LCN | 0.85 | 1.57 | 0.72 | 0.1 KOH | [20] |

**Table S6**. Comparison between the performance of rechargeable ZABs with FeNi-NC@MWCNTs and other recently reported ZABs with single/dual atom catalysts.

| Cathode Catalysts | Peak power density (mW $cm^{-2}$) | Specific capacity (mAh $g^{-1}$) | Stability test | Reference |
|---|---|---|---|---|
| FeNi-NC@MWCNTs | 218.3 | 781.7 @15 mA $cm^{-2}$ | 300 h @15 mA $cm^{-2}$ | This work |
| $FeMn_{ac}/Mn-N_4C$ | 207 | 720.2 @5 mA $cm^{-2}$ | 100 h @5 mA $cm^{-2}$ | [21] |
| FeCo-DACs/NC | 175 | - | 240 h @15 mA $cm^{-2}$ | [22] |
| Cu-Se DAs | 206.5 | 809.3 @10 mA $cm^{-2}$ | 70 h @10 mA $cm^{-2}$ | [23] |
| $Pd/WO_{2.72}$ | 168 | 633 @25 mA $cm^{-2}$ | - | [8] |
| Fe SAs@NC | 107.9 | 734.5 @10 mA $cm^{-2}$ | 183 h @10 mA $cm^{-2}$ | [24] |
| Fe-Zn-SA/NC | 167.2 | 756.6 @10 mA $cm^{-2}$ | 120 h @10 mA $cm^{-2}$ | [25] |
| Ru-SAS/SNC | 229 | 728 @10 mA $cm^{-2}$ | 270 h @5 mA $cm^{-2}$ | [26] |
| CoMn/NC | 176 | - | 22 h @5 mA $cm^{-2}$ | [27] |
| Fe,Co,N-C | 198.4 | 726 @2 mA $cm^{-2}$ | 51 h @5 mA $cm^{-2}$ | [15] |
| $Fe_1Co_1$-CNF | 201.7 | 814 @10 mA $cm^{-2}$ | 200 h @20 mA $cm^{-2}$ | [16] |
| SA&NP-FeCo-NTS | 102.2 | 770 @20 mA $cm^{-2}$ | 250 h @5mA $cm^{-2}$ | [28] |
| FeMn-DSAC | 184 | - | 80 h @2 mA $cm^{-2}$ | [29] |
| Fe-N/P-C-700 | 133.2 | 723.6 @100 mA $cm^{-2}$ | 40 h @10 mA $cm^{-2}$ | [30] |
| FeSA/N-PSCS | 164.5 | 725.3 @10 mA $cm^{-2}$ | 150 h @10 mA $cm^{-2}$ | [31] |
| FeCo SAs@Co/N-GC | 162 | 741 @5 mA $cm^{-2}$ | 300 h @5 mA $cm^{-2}$ | [32] |

## III. Author contributions:

C. Streb, R. Liu, Z. Chen conceived the idea, designed the experiments, planned synthesis, supervised the project, and revised the manuscript. Z. Chen designed the experiments, performed catalyst synthesis, and electrochemical measurements, analyzed the characterization results and wrote the manuscript. K. Cao and M. Jin carried out the TEM measurements. S. Rahali performed the ICP-OES tests. E. Ebrahimi performed the SEM measurements. W. Cheng and N. Ma conducted the DFT calculations and performed data analysis. B.J. Huang, K. Lakshmanan and S. A. Chala performed XAS analyses. C.-Y. Chang and C.C. Cheung performed XAS fitting. H. Luo, Y. Wang performed Raman analysis. All authors discussed the results, drew conclusions, and commented on the manuscript.

## IV. References:


[1] G. Kresse, J. Hafner, *Phys. Rev. B* **1994**, *49*, 14251–14269.

[2] G. Kresse, J. Furthmüller, *Comput. Mater. Sci.* **1996**, *6*, 15–50.

[3] D. Joubert, *Phys. Rev. B - Condens. Matter Mater. Phys.* **1999**, *59*, 1758–1775.

[4] J. P. Perdew, K. Burke, M. Ernzerhof, *Phys. Rev. Lett.* **1998**, *80*, 891.

[5] K. Lee, É. D. Murray, L. Kong, B. I. Lundqvist, D. C. Langreth, *Phys. Rev. B - Condens. Matter Mater. Phys.* **2010**, *82*, 3–6.

[6] Z. Wang, R. Xu, Q. Ye, X. Jin, Z. Lu, Z. Yang, Y. Wang, T. Yan, Y. Liu, Z. Pan, S. J. Hwang, H. J. Fan, *Adv. Funct. Mater.* **2024**, 2315376.

[7] W. Huang, B. Hai, G. Su, H. Mao, J. Li, *Mater. Lett.* **2024**, *360*, 135976.

[8] Z. Yang, N. Jiang, S. Bei, K. Bao, M. Xiang, C. Yu, S. Dong, H. Qin, *Electrochim. Acta* **2024**, *476*, 143768.

[9] W. H. Wang, C. H. Han, W. X. Hong, Y. C. Chiu, I. H. Tseng, Y. H. Chang, H. Pourzolfaghar, Y. Y. Li, *J. Energy Storage* **2024**, *85*, 111058.

[10] J. E. Tsai, W. X. Hong, H. Pourzolfaghar, W. H. Wang, Y. Y. Li, *Chem. Eng. J.* **2023**, *460*, 141868.

[11] J. Yan, M. Tian, R. Shi, T. Gu, K. Zeng, J. Zhou, Q. Zhang, M. H. Rümmeli, R. Yang, *Mater. Today Energy* **2022**, *30*, 101171.

[12] L. Gao, X. Gao, P. Jiang, C. Zhang, H. Guo, Y. Cheng, *Small* **2022**, *18*, 2105892.

[13] M. Liu, N. Li, S. Cao, X. Wang, X. Lu, L. Kong, Y. Xu, X. H. Bu, *Adv. Mater.* **2022**, *34*, 2107421.

[14] C. X. Zhao, J. N. Liu, J. Wang, C. Wang, X. Guo, X. Y. Li, X. Chen, L. Song, B. Q. Li, Q. Zhang, *Sci. Adv.* **2022**, *8*, eabn5091.

[15] S. Sarkar, A. Biswas, E. E. Siddharthan, R. Thapa, R. S. Dey, *ACS Nano* **2022**, *16*, 7890−7903.

[16] Y. Wang, Z. Li, P. Zhang, Y. Pan, Y. Zhang, Q. Cai, S. R. P. Silva, J. Liu, G. Zhang, X. Sun, Z. Yan, *Nano Energy* **2021**, *87*, 106147.

[17] K. Wang, Z. Lu, J. Lei, Z. Liu, Y. Li, Y. Cao, *ACS Nano* **2022**, *16*, 11944–11956.

[18] J. Chen, H. Li, C. Fan, Q. Meng, Y. Tang, X. Qiu, G. Fu, T. Ma, *Adv. Mater.* **2020**, *32*, 2003134.

[19] B. Guo, Q. Ju, R. Ma, Z. Li, Q. Liu, F. Ai, M. Yang, S. Kaskel, J. Luo, T. Zhang, J. Wang, *J. Mater. Chem. A* **2019**, *7*, 19355–19363.

[20] X. Li, Y. Liu, H. Chen, M. Yang, D. Yang, H. Li, Z. Lin, *Nano Lett.* **2021**, *21*, 3098–3105.

[21] H. Liu, L. Jiang, J. Khan, X. Wang, J. Xiao, H. Zhang, H. Xie, L. Li, S. Wang, L. Han, *Angew. Chemie* **2023**, *135*, 22–27.

[22] M. Liu, N. Li, S. Cao, X. Wang, X. Lu, L. Kong, Y. Xu, X. H. Bu, *Adv. Mater.* **2022**, *34*, 2107421.

[23] Z. Sun, H. Zhang, L. Cao, X. Liu, D. Wu, X. Shen, X. Zhang, Z. Chen, S. Ru, X. Zhu, Z. Xia, Q. Luo, F. Xu, T. Yao, *Angew. Chemie* **2023**, *135*, e202217719.

[24] W. J. Niu, Y. Y. Yan, R. J. Li, W. W. Zhao, J. L. Chen, M. J. Liu, B. Gu, W. W. Liu, Y. L. Chueh, *Chem. Eng. J.* **2023**, *456*, 140858.

[25] J. Xu, S. Lai, D. Qi, M. Hu, X. Peng, Y. Liu, W. Liu, G. Hu, H. Xu, F. Li, C. Li, J. He, L. Zhuo, J. Sun, Y. Qiu, S. Zhang, J. Luo, X. Liu, *Nano Res.* **2021**, *14*, 1374–1381.

[26] J. Qin, H. Liu, P. Zou, R. Zhang, C. Wang, H. L. Xin, *J. Am. Chem. Soc.* **2022**, *144*, 2197–2207.

[27] G. Dey, R. Jana, S. Saifi, R. Kumar, D. Bhattacharyya, A. Datta, A. S. K. Sinha, A. Aijaz, *ACS Nano* **2023**, *17*, 19155–19167.

[28] Q. Zhang, P. Liu, X. Fu, Y. Yuan, L. Wang, R. Gao, L. Zheng, L. Yang, Z. Bai, *Adv. Funct. Mater.* **2022**, *32*, 2112805.

[29] T. Cui, Y. P. Wang, T. Ye, J. Wu, Z. Chen, J. Li, Y. Lei, D. Wang, Y. Li, *Angew. Chemie - Int. Ed.* **2022**,

*61*, e202115219.

[30] K. Yuan, D. Lützenkirchen-Hecht, L. Li, L. Shuai, Y. Li, R. Cao, M. Qiu, X. Zhuang, M. K. H. Leung, Y. Chen, U. Scherf, *J. Am. Chem. Soc.* **2020**, *142*, 2404–2412.

[31] M. Shen, J. Liu, J. Li, C. Duan, C. Xiong, W. Zhao, L. Dai, Q. Wang, H. Yang, Y. Ni, *Energy Storage Mater.* **2023**, *59*, 102790.

[32] N. K. Wagh, D. H. Kim, S. H. Kim, S. S. Shinde, J. H. Lee, *ACS Nano* **2021**, *15*, 14683–14696.
